# Genome mapping coupled with CRISPR gene editing reveals a P450 gene confers avermectin resistance in the beet armyworm

**DOI:** 10.1371/journal.pgen.1009680

**Published:** 2021-07-12

**Authors:** Yayun Zuo, Yu Shi, Feng Zhang, Fang Guan, Jianpeng Zhang, René Feyereisen, Jeffrey A. Fabrick, Yihua Yang, Yidong Wu

**Affiliations:** 1 The Key Laboratory of Plant Immunity and College of Plant Protection, Nanjing Agricultural University, Nanjing, China; 2 Institute of Pesticide Science, College of Plant Protection, Northwest A&F University, Yangling, Shaanxi, China; 3 Department of Plant and Environmental Sciences, University of Copenhagen, Copenhagen, Denmark; 4 USDA ARS, U.S. Arid Land Agricultural Research Center, Maricopa, Arizona, United States of America; University of Kentucky, UNITED STATES

## Abstract

The evolution of insecticide resistance represents a global constraint to agricultural production. Because of the extreme genetic diversity found in insects and the large numbers of genes involved in insecticide detoxification, better tools are needed to quickly identify and validate the involvement of putative resistance genes for improved monitoring, management, and countering of field-evolved insecticide resistance. The avermectins, emamectin benzoate (EB) and abamectin are relatively new pesticides with reduced environmental risk that target a wide number of insect pests, including the beet armyworm, *Spodoptera exigua*, an important global pest of many crops. Unfortunately, field resistance to avermectins recently evolved in the beet armyworm, threatening the sustainable use of this class of insecticides. Here, we report a high-quality chromosome-level assembly of the beet armyworm genome and use bulked segregant analysis (BSA) to identify the locus of avermectin resistance, which mapped on 15–16 Mbp of chromosome 17. Knockout of the *CYP9A186* gene that maps within this region by CRISPR/Cas9 gene editing fully restored EB susceptibility, implicating this gene in avermectin resistance. Heterologous expression and *in vitro* functional assays further confirm that a natural substitution (F116V) found in the substrate recognition site 1 (SRS1) of the CYP9A186 protein results in enhanced metabolism of EB and abamectin. Hence, the combined approach of coupling gene editing with BSA allows for the rapid identification of metabolic resistance genes responsible for insecticide resistance, which is critical for effective monitoring and adaptive management of insecticide resistance.

## Author summary

Insecticide resistance is a global constraint to agricultural production, and rapid identification of resistance genes is critical for better monitoring and management of resistant insect pests. Identification of metabolic resistance genes has always been a challenging task due to the high diversity of insect detoxification enzyme genes. Here, we report a high-quality chromosome-level assembly of the beet armyworm genome and use bulked segregant analysis (BSA) to identify the locus of avermectin resistance, which mapped on 15–16 Mbp of chromosome 17. Knockout of the *CYP9A186* gene that maps within this region by CRISPR/Cas9 gene editing fully restored avermectin susceptibility. Heterologous expression and *in vitro* functional assays further confirm that a natural substitution (F116V) found in the substrate recognition site 1 (SRS1) of the CYP9A186 protein results in enhanced metabolism of avermectin. Hence, the combined approach of coupling gene editing with BSA allows for the rapid identification of metabolic resistance genes responsible for insecticide resistance, which is critical for field monitoring of such mutations, for making improved decisions on appropriate use of effective chemistries, as well for improvements in the design of future compounds that target *S*. *exigua*.

## Introduction

Avermectins are a group of 16-membered macrocyclic lactones possessing potent anthelmintic and insecticidal activities [1]. Emamectin benzoate (EB) [(4″R)-4″-deoxy-4″-(methylamino) avermectin B1 benzoate] is a macrocyclic lactone semi-synthetic derivative of the avermectin family and has much higher potency than abamectin against a number of lepidopterous pests [2]. Similar to abamectin and ivermectin, the mode of action of EB in invertebrates is through the overstimulation of glutamate-gated chloride channels (GluCls) [1–3]. With extensive field application of avermectins, the evolution of resistance in mites, insects, and parasitic worms has become an increasingly serious problem in agriculture [4–7].

At least two major mechanisms are known to cause avermectin resistance in arthropods: target-site insensitivity and metabolic enzyme-based resistance [8]. Target site mutations in the glutamate-gated chloride channels are associated with resistance to avermectins in *Drosophila melanogaster* [9], *Plutella xylostella* [10,11] and *Tetranychus urticae* [5,12]. Enhanced oxidative metabolism of abamectin has also been reported as a resistance mechanism in *Leptinotarsa decemlineata* [13], *Bemisia tabaci* [14], *P*. *xylostella* [15] and *T*. *urticae* [16]. Enhanced avermectin metabolism due to increased expression of cytochrome P450 was shown in *T*. *urticae* [17] and in *Pediculus humanus humanus* [3]. Other detoxification mechanisms, including sequestration and/or the metabolic action of esterases, carboxylesterases, and/or glutathione S-transferases (GSTs) may also play a role in avermectin resistance in some arthropods [16,18]. However, the underlying molecular mechanisms of resistance to EB and abamectin remain largely unknown in lepidopteran pests.

The beet armyworm, *Spodoptera exigua*, is an important global pest of many crops [6,19] and because of repeated overuse of EB, it has evolved high levels of insecticide resistance in the field in some parts of Asia, including Pakistan and China [6,19]. Bulk segregant analysis (BSA) has recently been used to rapidly identify genomic regions associated with unique phenotypes in numerous different species [20]. To better understand the molecular genetic basis of EB resistance, we first produced a chromosome-level *de novo* genome assembly from a EB-susceptible reference beet armyworm laboratory strain from China. We then used BSA to identify loci containing major resistance genes and used CRISPR/Cas9 gene-editing to map resistance to the *CYP9A186* gene. Furthermore, the natural substitution (F116V) in the substrate binding site 1 (SRS1) region of CYP9A186 was functionally verified to confer resistance to both avermections, EB and abamectin. Hence, the combined use gene editing with BSA provided a rapid and highly efficient means to identify metabolic resistance genes responsible for insecticide resistance. Such results are critical for the monitoring of field populations of the beet armyworm for avermectin resistance and for the future use and/or design of new chemistries to manage resistant populations.

## Results

### Chromosome-level genome assembly

**Fig 1:**
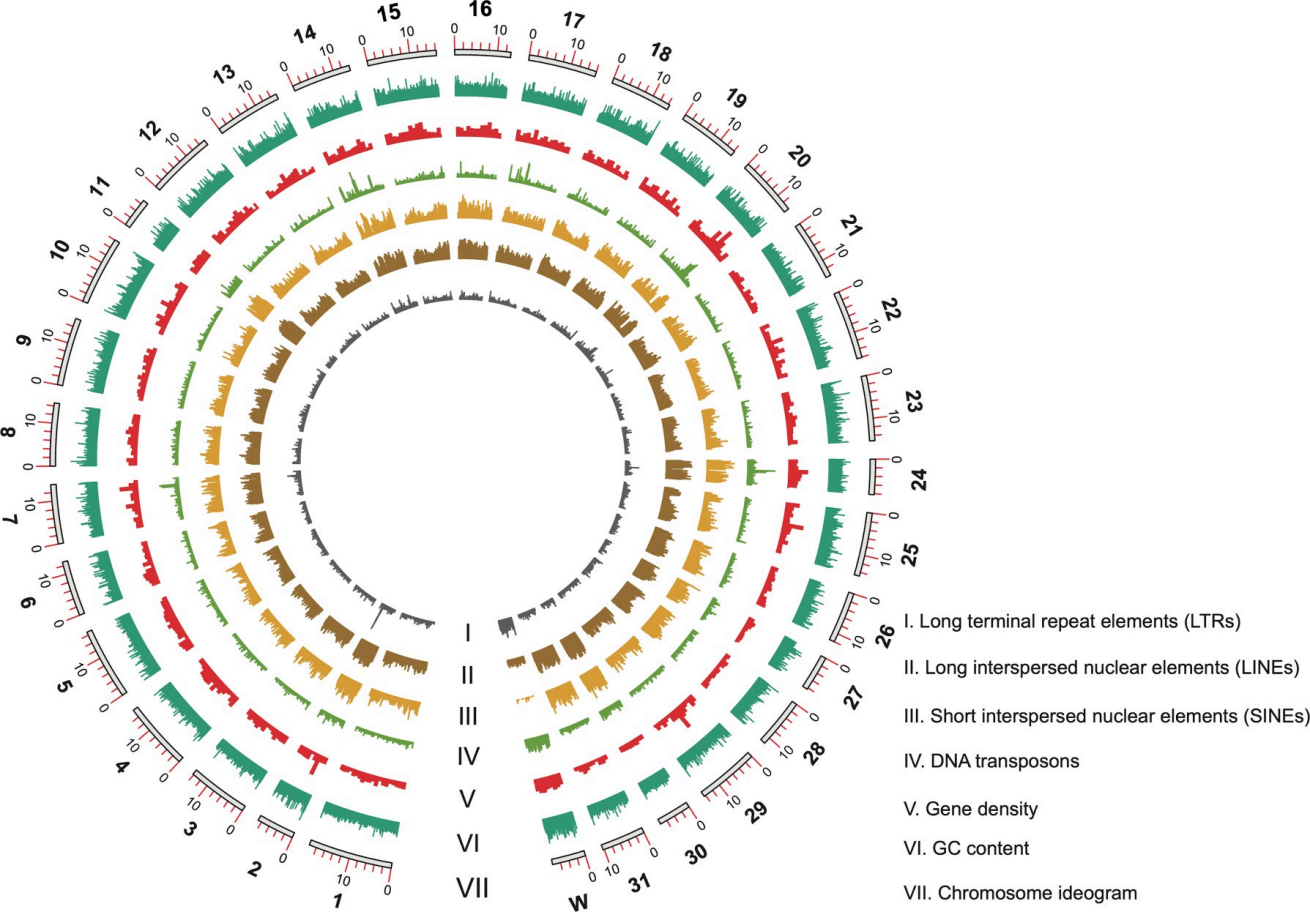
Circos plot of beet armyworm genome. Tracks from innermost to outermost are: I. Long terminal repeat elements (LTRs), II. Long interspersed nuclear elements (LINEs), III. Short interspersed nuclear elements (SINEs), IV. DNA transposons, V. Gene density, VI. GC content, and VII. Chromosome ideogram with numbers indicating the chromosome nomenclature order and size (in Mb). A 10 kb sliding window is shown for the GC content track and 100 kb for all other tracks.

To facilitate genetic mapping of resistance, we first sequenced the beet armyworm genome using both Illumina and PacBio whole genome sequencing (WGS). We generated 3,919,515 PacBio subreads yielding 38.98 Gb (87X coverage) and 56.32 Gb of Hi-C sequencing data (126X coverage), respectively. Estimated genome size ranged from 408.58 Mb to 448.90 Mb with a low heterozygosity for the sequenced susceptible WH-S strain (**S1 Table**). The assembled genome consisted of 667 contigs spanning 446.80 Mb with a scaffold/contig N50 length of 14.36/3.47 Mb (**Table 1**); among them, 367 contigs were anchored into 32 pseudo-chromosomes (**Fig 1**), accounting for greater than 96% (429.74 Mb) of the genome. Consistent with lepidopteran females being heterogametic for sex chromosomes [21], 30 autosomes plus two sex chromosomes (Z and W) were identified from the sequenced female pupa. When compared with the insect_odb9 reference dataset consisting of 1,658 functional genes, BUSCO completeness analyses showed our final genome assembly was 97.9% complete, with only 0.2% fragmented and 1.9% missing BUSCO genes (**S2 Table**). Reflecting the high degree of completeness and accuracy of the genome assembly, we mapped 97.62% Illumina short reads and 93.78% of the PacBio long reads to our final assembly with very low redundancy (2.1% duplicated BUSCOs) (**Table 2**). Among four Noctuidae pest species (**Table 1**), assemblies of *S*. *exigua* and *S*. *frugiperda* have the highest genome contiguity (contig N50 > 1 Mb, contig number < 1,000) and the lowest gap content (< 0.1%).

**Table 1:** Genome assembly metrics for four Noctuidae species.

	*S*. *exigua*	*S*. *litura*^*a*^	*S*. *frugiperda*^*a*^	*H*. *armigera*^*a*^
**Accession number**	GCA_011316535.1	GCA_002706865.1	GCA_011064685.1	GCA_002156985.1
**Assembly size (Mb)**	446.80	438.96	486.29	337.09
**Number of scaffolds**	301	2,975	92	998
**Longest scaffold (Mb)**	19.74	18.94	22.37	6.15
**Scaffold N50 length (Mb)**	14.36	13.59	16.35	1.00
**Number of contigs**	667	13,637	617	24,552
**Longest contig (Mb)**	15.88	0.64	16.14	0.31
**Contig N50 length (Mb)**	3.47	0.068	1.13	0.023
**Pseudo-chromosomes**	32	31	32	31
**GC (%)**	36.67	36.56	36.43	37.50
**Gaps (%)**	0.075	2.49	0.011	11.01
**BUSCO completeness (%)**	97.9	97.1	93.4	96.8

^a^ Reference sources: *Spodoptera litura* [22], *Spodoptera frugiperda* [87] and *Helicoverpa armigera* [88,89].

**Table 2:** Genome annotation statistics of the beet armyworm.

Elements	Number
Protein-coding genes	17,727
Mean protein (aa) and gene (bp) length	482.19/9,604.99
Exons/introns per gene	6.40/5.20
Exon	37.95 Mb (8.49%)
Intron	132.31 Mb (28.34%)
Mean exon/intron length	334.78/1,434.55
BUSCO completeness (%)	96.6
GO	10,298
Reactome pathway	2,731
KEGG orthology	7,643
KEGG pathway	4,493
EC	2,675
COG	12,987

### Gene annotation

To comprehensively annotate genes in our assembled beet armyworm genome, we integrated *ab initio*, transcriptome- and protein homology-based strategies to predict 17,727 protein-coding genes (e.g., gene models) with an overall mean length of 9,605 bp (**Table 2**). BUSCO assessment of the gene models positively identified 1,601 of the 1,658 reference genes (96.6%). Of these genes, only 45 (2.7%) were duplicated, 12 (0.7%) were fragmented, and 45 (2.7%) were missing, indicating the assemble genome is well-represented by its protein-coding genes. Comparison of the genome with RNA-seq data indicated a total mean exon and intron count of 6.4 and 5.2 per gene, with a mean length of 335 bp and 1,435 bp, respectively (**Table 2**). Among the predicted genes, 82% had transcriptome support and 96% had matches within the UniProt database. InterProScan identified protein domains for 13,186 (74%) genes, GO terms for 8,227 (47%) genes and Reactome pathways for 2,731 (15%) genes, respectively. EggNOG predicted GO terms for 6,706 genes, KEGG orthology (KO) matches for 7,643 genes, KEGG pathway matches for 4,493 genes, EC matches for 2,675 genes, and COG matches for 12,987 genes (**Table 2**).

Using *de novo* prediction and library alignments to detect repetitive DNA, we found that 33% (147.97 Mb) of the beet armyworm genome corresponds to putative transposable elements (TEs). This is consistent with the reported repeat content (32%) published for *S*. *litura* [22]. Several families of conserved TEs were found in the beet armyworm genome, including long interspersed nuclear elements (LINEs, 15%), rolling-circle transposition (RC, 4%), long terminal repeat elements (LTR, 3%), short interspersed nuclear elements (SINE, 3%), and DNA transposons (2%) (**S3 Table**). Simple repeats also occupied approximately 1% of the genome (**S3 Table**). Consistent with other lepidopterans [21], the beet armyworm chromosome W is enriched with DNA transposons and LTR retrotransposons when compared with the other 30 automosomes and the Z chromosome (**Fig 1**).

### Bulked segregant analysis

**Fig 2:**
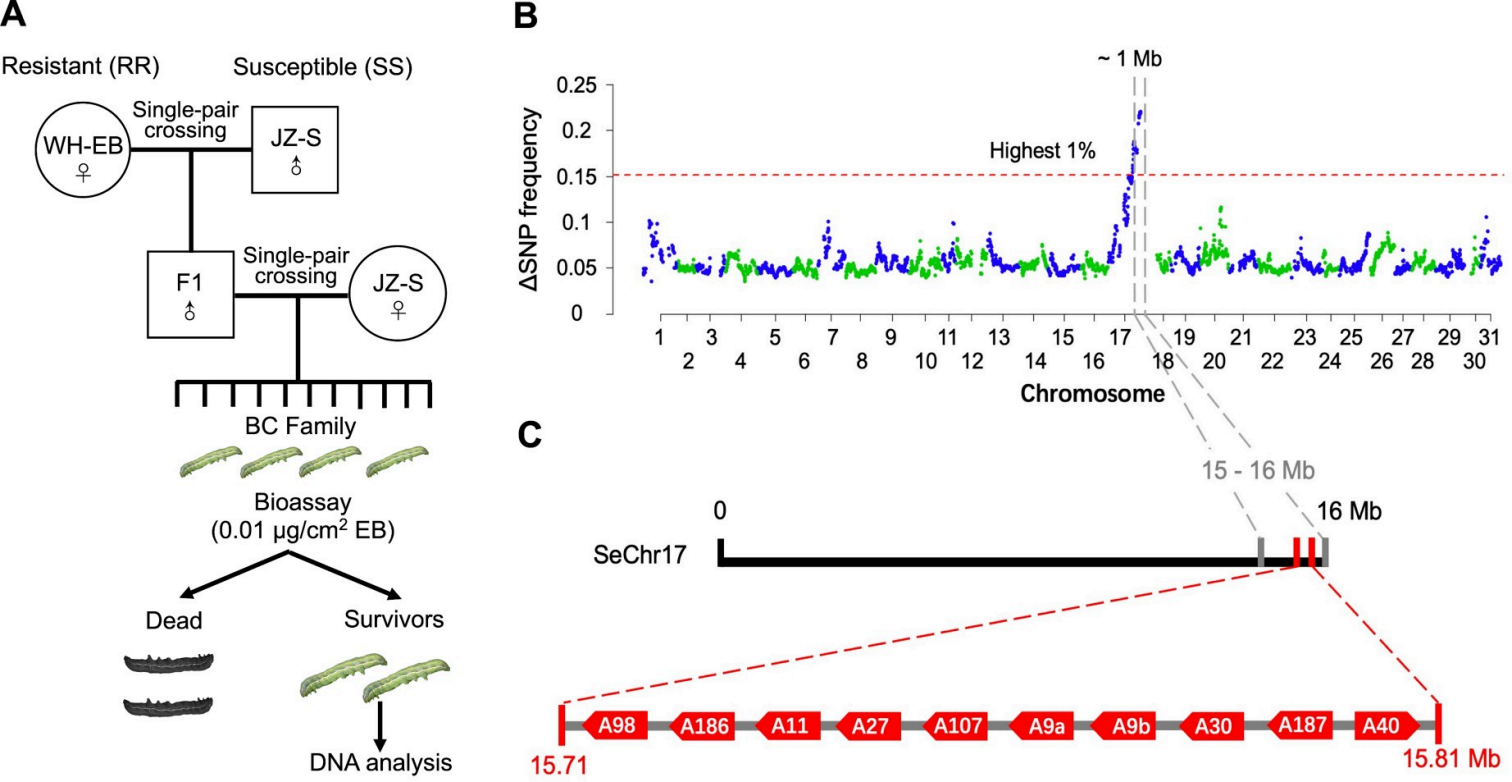
Experimental design for crosses, bioassays and bulked segregant analysis (BSA) to map emamectin benzoate resistance. (**A**) Single-pair crosses between JZ-S and WH-EB virgin adults produced families of hybrid F_1_ offspring. A male F_1_ was backcrossed to a female JZ-S to produce the backcross (BC) family. Progeny from the BC family were exposed to a diagnostic concentration of EB of which 120 survivors and their F_1_ parents were analyzed by next-generation sequencing (NGS). (**B**) BSA mapping revealed a 1 Mb genomic region in chromosome 17 highly correlated with EB resistance in WH-EB. The mean frequency deviation values obtained from a sliding window analysis (1 Mb window with 100 Kb step) were plotted across all 31 chromosomes of the genome. The red dashed line corresponds to the top 1% threshold of the SNP-index likely containing changes linked with EB resistance. (**C**) Expanded map of chromosome 17 (from 15 Mb to 16 Mb) that includes the *CYP9A* gene cluster (15.71 Mb to 15.81Mb).

Tissue samples (120 moth legs) from EB-resistant individuals (survivors) derived from a backcrossed family generated by crossing a resistant WH-EB female with a susceptible JZ-S male (**Fig 2A**) were pooled and used for bulked sequence analysis (BSA). In doing so, we identified a 1 Mb genomic region in chromosome 17 (from 15–16 Mb) that was significantly biased with resistance (**Fig 2B**). A cluster (chromosomal locations: 15.71 Mb to 15.81Mb) of *CYP* genes belonging to the CYP9A family is located in this region of chromosome 17 (**Fig 2C**), whose functions are frequently associated with xenobiotic detoxification [22–25]. Detailed analysis of the *CYP9A* gene cluster from the genome of *S*. *exigua* identified ten *CYP9A* genes, which were named *CYP9A40*, *CYP9A187*, *CYP9A30*, *CYP9A9a* and *CYP9A9b* (duplication), *CYP9A107*, *CYP9A27*, *CYP9A11*, *CYP9A186* and *CYP9A98* according to the International P450 Nomenclature Committee (**Fig 2C**). We therefore hypothesize that the dominant EB resistance in WH-EB is associated with one or more P450 genes in the *CYP9A* gene cluster of WH-EB.

### Genetic mapping of resistance using CRISPR/Cas9

**Fig 3:**
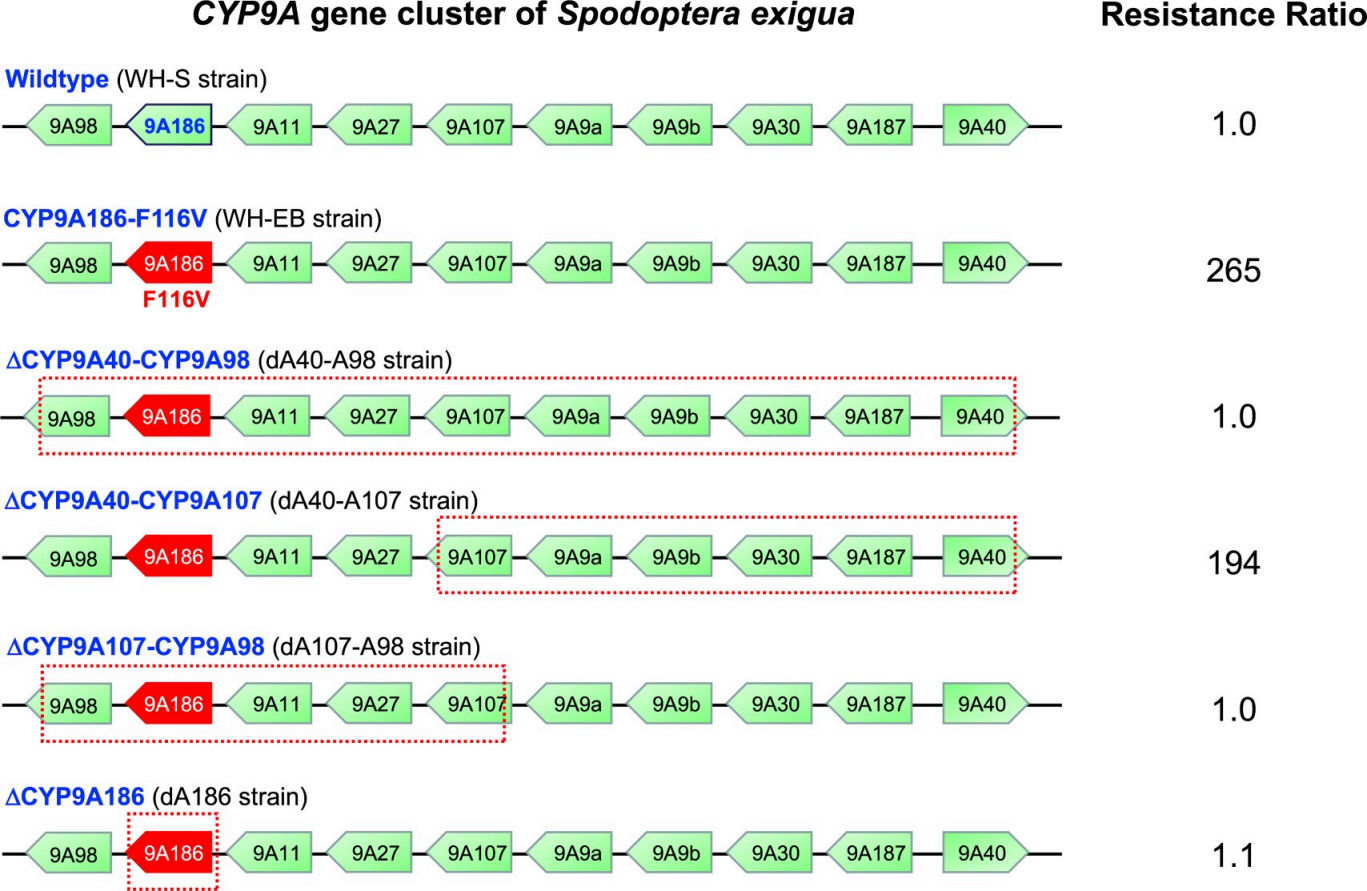
Genome mapping of the *CYP9A* gene cluster by CRISPR/Cas9 gene editing. Deletions in the *CYP9A* gene cluster are indicated with dotted boxes and include ΔCYP9A40-CYP9A98, ΔCYP9A40-CYP9A107, ΔCYP9A107-CYP9A98, and ΔCYP9A186. Resistance ratios to EB for each of the homozygous strains harboring both the natural CYP9A186-F116V mutation in resistant WH-EB strain and the CRISPR/Cas9-edited strains are shown.

To map resistance in the WH-EB strain, we created four homozygous knockout strains (dA40-A98, dA40-A107, dA107-A98, and dA186) and observed their corresponding resistance phenotypes (**Fig 3**). The first knockout of the entire CYP9A40 to CYP9A98 gene cluster (ΔCYP9A98-CYP9A40) resulted in a strain (e.g., dA40-A98) with complete restoration of susceptibility to EB (**Table 3**), indicating that at least one resistance gene is located in this region. Subsequent CRISPR/Cas9 knockout of the chromosomal fragment corresponding to CYP9A40 to CYP9A107 (ΔCYP9A107-CYP9A40) resulted in a strain (dA40-A107) that remained fully resistant to both insecticides (**Table 3**). In contrast, susceptibility was fully restored in the dA107-A98 strain where the chromosome fragment from CYP9A107 to CYP9A98 (ΔCYP9A107-CYP9A98) was deleted (**Table 3**), further narrowing the resistance gene to this region. Finally, we used CRISPR/Cas9 to generate a strain (dA186) homozygous for a 4-bp deletion in *CYP9A186* (ΔCYP9A186) that results in the introduction of a premature stop codon and the loss of downstream active sites within the cytochrome P450 enzyme (**S1 Fig**). Bioassays show that susceptibility to abamectin and EB was fully restored in the dA186 strain (**Table 3 and Fig 3**), confirming that resistance maps to the *CYP9A186* gene.

**Table 3:** Response to abamectin and emamectin benzoate of beet armyworm larvae from a susceptible strain (WH-S), a resistant strain (WH-EB), and four CRISPR/Cas9 gene-edited *CYP9A* knockout strains (dA40-A98, dA40-A107, dA107-A98, and dA186).

Insecticide	Strain	Slope	LC_50_ (μg/cm^2^)	95% Fiducial limits	RR^a^
Abamectin	WH-S	3.06±0.44	0.31	0.25–0.44	-
	WH-EB	2.60±0.36	13.89	10.86–19.10	45
	dA40-A98	2.53±0.34	0.26	0.20–0.35	0.8
	dA40-A107	2.26±0.33	15.72	11.93–22.93	51
	dA107-A98	4.23±0.66	0.45	0.37–0.56	1.4
	dA186	3.92±0.59	0.52	0.43–0.68	1.7
Emamectin benzoate	WH-S	2.57±0.35	0.0017	0.0014–0.0022	-
	WH-EB	3.39±0.48	0.45	0.37–0.58	265
	dA40-A98	3.33±0.51	0.0018	0.0014–0.0023	1.0
	dA40-A107	2.60±0.33	0.33	0.26–0.43	194
	dA107-A98	3.37±0.56	0.0017	0.0014–0.0021	1.0
	dA186	2.46±0.33	0.0019	0.0015–0.0024	1.1

^a^ Resistance ratio = LC_50_ (WH-EB or knockouts) / LC_50_ (WH-S).

### Identification of a natural mutation in *CYP9A186* from WH-EB associated with avermectin resistance

**Fig 4:**
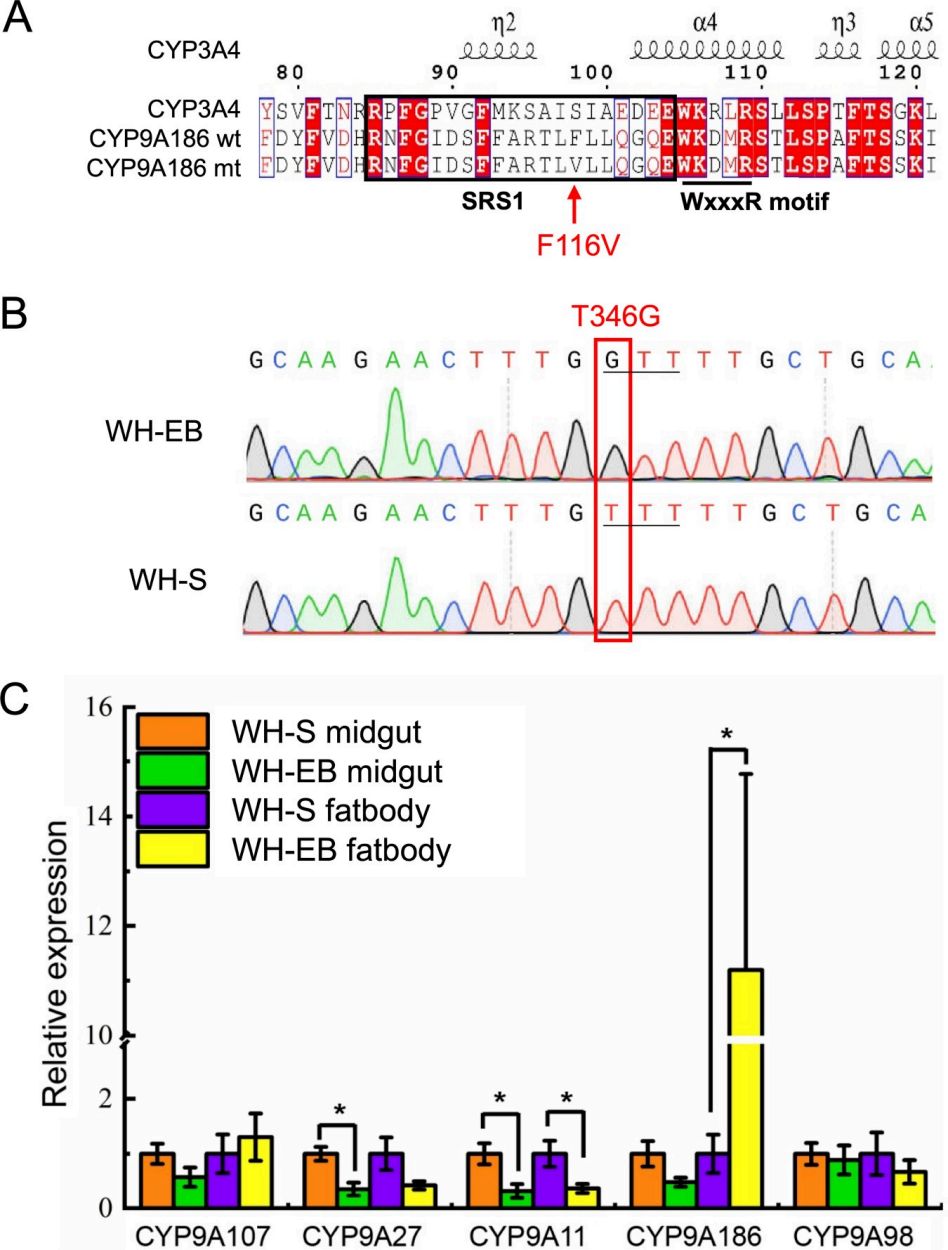
Identification of the *CYP9A186* T346G mutation resulting in the F116V substitution and quantitative real-time PCR transcriptional analysis of *CYP9A186*. (**A**) Alignment of the deduced CYP9A186 amino acid sequences from WH-EB and WH-S with a translated human cytochrome P450 (e.g., CYP3A4). The F116V substitution is shown with a red arrow. The predicted locations of alpha helical secondary structures are marked based on the template of the CYP3A4 protein structure (1TQN) using ESPript 3.0. (**B**) Representative chromatograms from direct sequencing of *CYP9A186* PCR products from WH-EB and WH-S showing the T346G mutation. (**C**) qRT-PCR analysis of *CYP9A107*, *CYP9A27*, *CYP9A11*, *CYP9A186* and *CYP9A98* in midgut and fat body from WH-S and WH-EB. Data shown represent means ± SE derived from four biological replicates. Asterisks indicate significant differences between the strains (Student’s *t*-test, *p*<0.05).

To determine if one of the P450 genes in the defined region associated with avermectin resistance differed between susceptible and resistant strains, we PCR-amplified, cloned and sequenced the full-length cDNAs corresponding to the open reading frames (ORF) of CYP9A107, CYP9A27, CYP9A11, CYP9A186 and CYP9A98 from WH-S and WH-EB, respectively. We found a single T346G mutation resulting in the F116V substitution within the SRS1 region of CYP9A186 (GenBank accession no. MN179472) in WH-EB and not WH-S (**Fig 4A**). DNA based genotyping of 20 larvae from each strain further revealed that WH-EB is homozygous for Val (GTT) at codon 116, whereas WH-S is homozygous for Phe (TTT) (**Fig 4B**). No other consistent mutations were found in the other four genes. Additionally, we also observed a 10-fold increase in *CYP9A186* transcript abundance (i.e., overexpression) in fat body tissue from WH-EB compared with WH-S (**Fig 4C**). None of the other four P450 candidate genes showed higher transcription in WH-EB compared to WH-S (**Fig 4C**). These data therefore show that a naturally-occurring point mutation and/or overexpression of *CYP9A186* is associated with avermectin resistance in WH-EB strain and provides additional supports for its involvement in metabolic insecticide resistance in the beet armyworm.

### Heterologous expression of P450s in cultured insect cells

Both wild-type (CYP9A186wt) and mutant (CYP9A186-F116V) CYP9A186 enzymes were expressed in *Trichoplusia ni* High Five (Hi5) cells using a baculovirus system. Reduced CO-difference spectra of CYP9A186wt and CYP9A186-F116V in purified recombinant microsomes showed a maximum peak near 450 nm (**S2 Fig**), indicating successful expression of functional enzymes within the Hi5 cells. In addition, both recombinant P450s showed O-debenzylation activities to 7-benzyloxy-4-trifluoromethyl coumarin (BFCOD), again indicating that the recombinant enzymes had correct folding and were active. Microsomes with CYP9A186wt and CYP9A186-F116V had BFCOD specific activities of 0.083 ± 0.001 and 0.057 ± 0.003 pmol/min/pmol P450, respectively (mean values ± SEM, n = 4). Non-insertion control Hi5 microsomes showed no BFCOD activity.

### EB and abamectin metabolism and identification of their metabolites

**Fig 5:**
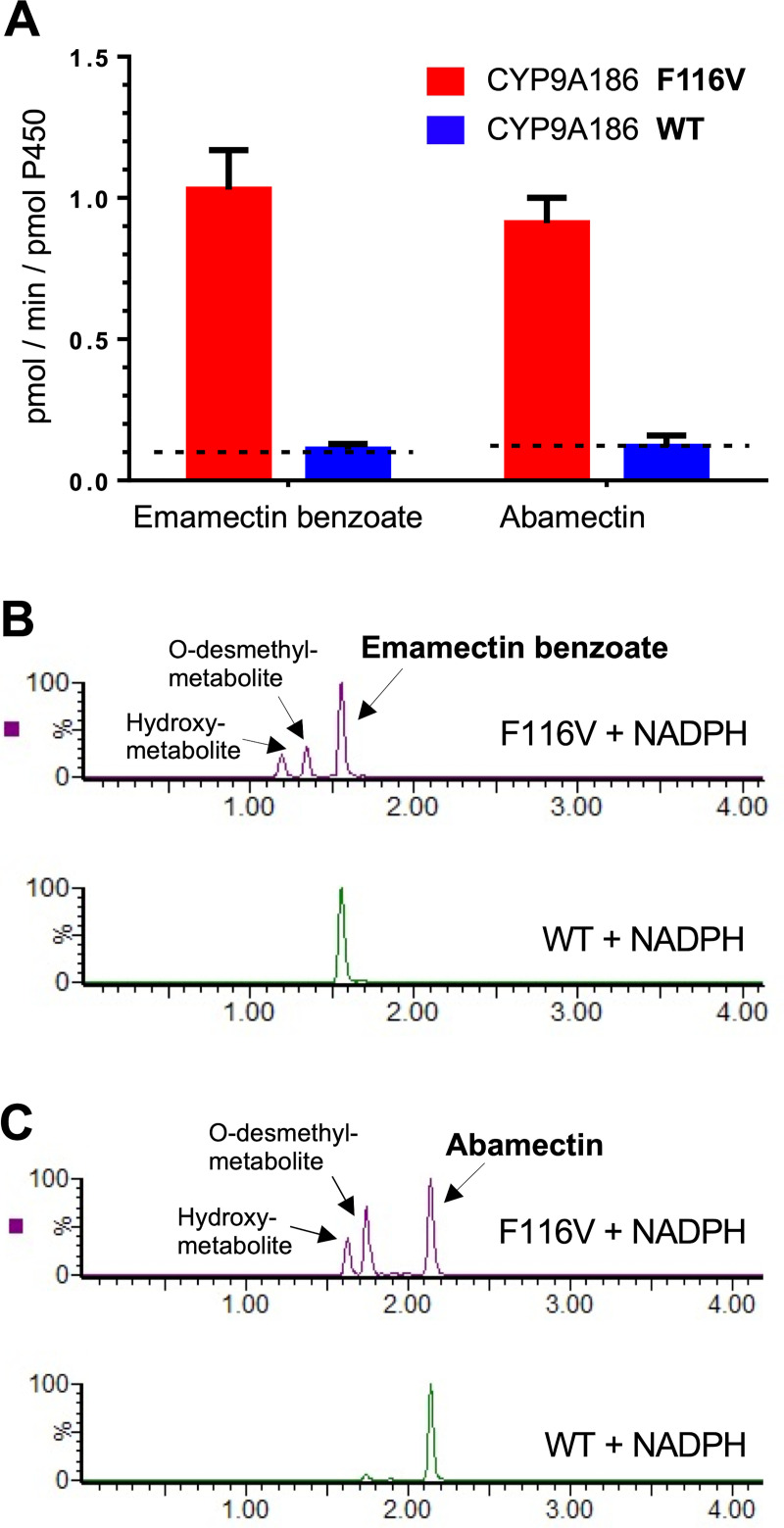
Metabolism of emamectin benzoate and abamectin by microsomes containing recombinant CYP9A186 and CYP9A186-F116V. (**A**) Specific activity (pmol/min/pmol P450) of EB and abamectin hydrolysis. Data are mean values ± SEM (n = 6). Dotted lines show mean limits of detection (LOD). There is no significant difference between WT and LOD (One sample *t*-test, p = 0.69 and 0.83 for emamectin benzoate and abamectin, respectively). (**B**) MRM signals of emamectin benzoate and its metabolites. (**C**) MRM signals of abamectin and its metabolites.

EB, abamectin and their multiple metabolites showed excellent separation and linearity under our experimental conditions (**Figs 5 and S3**). Recovery rates for both EB and abamectin were nearly 100% for the non-insertion control samples. Our *in vitro* assays showed that EB and abamectin were both metabolized by CYP9A186-F116V microsomal fractions at higher rates than by CYP9A186wt microsomes (**Fig 5A**). Whereas CYP9A186-F116V microsomes metabolized EB and abamectin at 1.03 ± 0.14 pmol/min/pmol P450 and 0.91 ± 0.09 pmol/min/pmol P450, respectively, the metabolic activity of both insecticides by CYP9A186wt microsomal fractions was negligible (0.11 ± 0.02 pmol/min/pmol P450 for EB and 0.12 ± 0.04 pmol/min/pmol P450 for abamectin). Indeed, specific activities of CYP9A186wt were not significantly higher than the limits of detection (LOD) for EB or abamectin (0.10 ± 0.01 pmol/min/pmol P450 and 0.13 ± 0.02 pmol/min/pmol P450, respectively) (**Fig 5A**). We observed no decrease of either EB or abamectin with microsomal protein fractions from cells infected with “non-insertion” control virus.

Two distinct metabolites were found in the CYP9A186-F116V microsomal reactions with EB (1.22 min and 1.37 min) and abamectin (1.65 min and 1.77 min) (**S3 Fig**). These metabolites were identified as (24’ or 26’) hydroxy-metabolites and O-desmethyl-metabolites by high resolution mass spectroscopy showing a quasi-molecular ion of m/z 902.5249 (hydroxy-EB, error = -1.3ppm, M + H), 872.5162 (O-desmethyl-EB, error = 0.8ppm, M + H), 906.5217 (hydroxy-abamectin, error = 0.8 ppm, M + NH_4_), 876.5096 (O-desmethyl-abamectin, error = -0.9 ppm, M+NH_4_) and a specific isotope peak ratio (n: n+1: n+2: n+3 = 10: 5: 1: 0.3) (**S4 Fig**).

These distinct hydroxy- and O-desmethyl-metabolites were only found in CYP9A186-F116V samples containing NADPH (**S3A and S3D Fig**), as the Ultra performance liquid chromatography-tandem mass spectrometer (UPLC-MS/MS) multireaction monitoring (MRM) signals corresponding to these two metabolites were not above the noise signal for the CYP9A186wt reaction samples (**S3B and S3E Fig**). These results confirm that unlike the CYP9A186-F116V samples, CYP9A186wt does not metabolize EB or abamectin under our experimental conditions.

## Discussion

The results reported here show the molecular genetic and biochemical basis of resistance to EB and abamectin in a laboratory-selected strain of beet armyworm from China. We first produced a chromosome-level *de novo* genome assembly and used BSA to identify the *CYP9A* gene cluster as the major locus associated with EB resistance. We then coupled CRISPR/Cas9 gene-editing to knockout regions within this gene cluster and mapped resistance to the *CYP9A186* gene. Heterologous expression of recombinant CYP9A186 tested with *in vitro* metabolism assays confirmed that a natural mutation (T346G), resulting in the F116V substitution located in the substrate binding site 1 (SRS1) region of CYP9A186, causes resistance to both EB and abamectin. Hence, the combined use of CRISPR/Cas9 gene editing with BSA provided a rapid and highly efficient means to identify metabolic resistance genes responsible for avermectin insecticide resistance in the beet armyworm.

The evolution of pesticide resistance is an increasingly intractable problem affecting crop production worldwide [26]. To combat resistance, there is an urgent need for the rapid identification of not only markers for resistance, but also genes that cause pesticide resistance. Numerous studies point to target-site resistance and metabolic resistance as the two primary mechanisms by which insects evolve resistance to insecticides [27,28]. Target-site resistance involves alterations (e.g., mutations) in the insecticide target protein that reduce its sensitivity to insecticides [29]. For many insecticides, the primary insecticide targets are known, and identification of mutations can be directly assessed. In contrast, metabolic resistance can involve numerous different pathways consisting of unrelated gene products, making their identification difficult due to the high diversity of detoxification enzyme genes in insect pests [30,31].

One efficient way to overcome the above problem is to map resistance genes within finite regions of insect genomes by BSA [32]. To do this, a high-quality genome map is an absolute prerequisite for precise genetic localization. Hence, we generated a chromosome-level assembly of the beet armyworm genome by single-molecule real-time PacBio and Hi-C sequencing. Even with a precise genomic map, BSA can often be difficult to identify specific and functional genes responsible for the observed phenotype. Improvements in BSA will likely come as sequencing technologies and methods are further developed to increase their accuracy and shorten the confidence interval of genetic mapping, especially in regions of chromosomes dense with gene clusters and/or with low recombination rates. In addition to increased sequencing resolution, methods to improve how genetic loci identified by BSA are linked to resistance phenotypes via functional genomics are needed. Recently, the CRISPR/Cas9 genome editing system was shown to be effective in several lepidopterans, including *S*. *exigua* [33–35], providing a powerful functional genomics tool for the exploration of the complex biology of insecticide resistance. Here, we show for the first time that BSA used in conjunction with CRISPR/Cas9 gene editing allows for the specific and rapid identification of a gene responsible for metabolic insecticide resistance. Such information is important for tracking resistant beet armyworm individuals in the field and for making better informed decisions about the appropriate use of avermectin and related insecticides to delay the further spread of resistance. Furthermore, the identification of specific mutations involved in the structure and detoxification function of CYP9A186 may enable the rational design of new and more potent chemistries that target pest insects.

A P450 gene, CYP392A16, is highly over-expressed in the abamectin resistant Mar-ab strain of *T*. *urticae*, and this P450 can metabolize abamectin *in vitro* [17]. However, P450 point mutations that increase the metabolism of avermectins have not been previously reported. For cytochrome P450s, six substrate recognition sites (SRSs) are key to the acquisition of novel functions [36]. Site-directed mutagenesis studies previously showed that SRS1, SRS4, SRS5, and SRS6 possess amino acid residues that form the catalytic site, while SRS2 and SRS3 participate in the formation of the substrate access channel [37]. Conservative amino acid substitutions in these SRS regions can alter P450 activities and/or substrate profiles, and some substitutions in non-SRS regions on the proximal surface of a P450 can also improve kinetic properties due to alterations in interactions with the P450 redox partners [37]. Here, we show that the single amino acid substitution F116V in SRS1 of CYP9A186 is a gain-of-function mutation that enables enhanced metabolism of EB and abamectin. Although this Phe to Val substitution appears to be relatively minor, the replacement of the bulky phenyl aromatic ring for the isopropyl moiety apparently has a major affect on the CYP9A186 active site. Indeed, CYP9A186 belongs to the CYP3 clan of cytochrome P450s, which contains other well-characterized P450s, including the human CYP3A4 whose three-dimensional structure has been solved [38]. Alignment of CYP9A186 with CYP3A4 (**Fig 4**) shows that F116 in CYP9A186 likely corresponds with S119 of CYP3A4 within the B’-C loop. This residue likely protrudes into the active site, where it is in close proximity to the heme and opposite the C-terminal loop, allowing for direct interaction with substrate [38,39]. We speculate that the F116V substitution could reduce structural hindrance within the active site and allow for acceptance of a broader suite of substrates hydroxylated by the P450.

Changes in P450 transcription as well as structural changes appear to alter insecticide susceptibility and must be evaluated on a case-by-case basis. Recently, CYP321A8 was shown to contribute resistance to pyrethroid and organophosphate insecticides in *S*. *exigua* through overexpression regulated by cis- and trans-regulatory elements [40]. In *D*. *melanogaster*, transgenic overexpression of *CYP6G2* leads to resistance to diazinon [41], whereas changes in transcript abundance was not involved for the closely related *CYP6G1*. It was rationalized that CYP6G2 has multiple hydrophobic substitutions that increased the size of its catalytic site along with one positive-to-polar substitution that eliminated a potentially critical charge in SRS2 [37,42]. Similarly, structural changes in a P450 active site are important in *Anopheles gambiae*, where CYP6Z1 metabolizes DDT but CYP6Z2 does not [43,44]. Overlays of the predicted CYP6Z1 and CYP6Z2 structures indicated that R210 (SRS2), I298 and E302 (SRS4) of the CYP6Z2 protrude sufficiently enough into the CYP6Z2 catalytic site that it could no longer dock large hydrophobic substrates such as DDT [37]. In *Anopheles funestus*, alleles of *CYP6P9b* that confer high pyrethroid resistance are characterized by three point mutations, as well as constitutive overexpression [45]. More recently, Zimmer et al. [46] reported several point mutations were associated with gene duplications for CYP6ER1 gene variants of imidacloprid resistant *Nilaparvata lugens* strains. When two SRS5 mutations (A375del and A376G) were tested in transgenic *Drosophila*, imidacloprid resistance increased 20-fold. A similar level of resistance was obtained with the T318S (in SRS4) substitution. When this T318S substitution was combined with both A375del and A376P, this combination exhibited the highest resistance of all transgenic lines (35-fold). Such examples indicate that variation in both SRS regions and non-SRS regions of CYPs contribute to differences in metabolic capability and that gain-of-function mutations are common for several classes of insecticides.

*In vivo* and *in vitro* metabolism studies in vertebrates have identified three major oxidative metabolites of abamectin: 24-hydroxymethyl (24-OH), 26-hydroxymethyl (26-OH), and 3’-O-desmethyl abamectin [47]. In our study, CYP9A186-F116V also catalyzes the hydroxylation and O-demethylation of EB and abamectin, most likely also leading to the (24’ or 26’) hydroxy-metabolites and 3’-O-desmethyl-metabolites. In contrast, CYP392A16 from *T*. *urticae* did not O-demethylate abamectin, but rather catalyzed the hydroxylation of the insecticide to produce metabolites that were less toxic to *T*. *urticae* than the parental compound [17].

Alleles with fitness costs are known to have a decreased ability to survive and reproduce in the absence of the insecticide [48]. Previous research reported that the WH-EB strain of *S*. *exigua* developed 1,110-fold resistance to EB and that two or a few genes contributed to resistance [49]. Subsequently, the WH-EB strain was reared without further selection with EB. This strain now maintains a relatively stable resistance level (~300 fold) to EB. It is therefore likely that one or a few minor genes that contributed to resistance in the WH-EB strain were lost due to fitness costs, and only the mutated CYP9A186 with little or no associated fitness costs, was retained.

Here, our combined approach of coupling gene editing with BSA allowed for the rapid identification of *CYP9A186* as the primary gene involved in metabolic resistance to both EB and abamectin in a strain of the beet armyworm from China. The accuracy and speed at which we identified, mapped, and validated the functional role of *CYP9A186* suggests that such methods are highly adoptable for the rapid identification of resistance in many arthropod pest/insecticide systems. We can further use this information to proactively track beet armyworm avermectin resistance in field populations and make better informed decisions on the use of appropriate chemistries to further delay the evolution of insecticide resistance. Our findings also further enhance the understanding of avermectin resistance evolution and suggests that gain-of-function mutations in the SRS regions of P450 are important for the acquisition of novel detoxification functions. As such, future work to monitor *CYP9A186* for mutations in the field as well the design of specific compounds to target/inhibit *CYP9A186* could be important for resistance management of *S*. *exigua*.

## Materials and methods

### Insects

We used three strains of beet armyworm from China: WH-S, JZ-S, and WH-EB. WH-S is a avermectin-susceptible strain was obtained from Wuhan Institute of Vegetables (Hubei, China) in 2009 and has been maintained in the laboratory for more than 20 years without exposure to any insecticides [6]. JZ-S is a susceptible strain obtained from the College of Plant Science and Technology, Huazhong Agriculture University (Wuhan, China) in 2018 and has been maintained in laboratory without exposure to any insecticides since its initial collection from Jingzhou, Hubei Province of China in 2003. WH-EB was derived from a field-collected strain with high levels of resistance to EB and is a near-isogenic strain introgressed with WH-S [49]. WH-EB was maintained without selection with EB since 2015.

### Genome sequencing

For genome sequencing, genomic DNA (gDNA) was extracted from two female pupae from the WH-S strain, including one pupa for both Illumina and PacBio whole genome sequencing (WGS) and the other pupa for Hi-C sequencing. gDNA was extracted using the Blood & Cell Culture DNA Mini Kit from Qiagen (Hilden, Germany). Long read libraries were constructed using a SMRTbell DNA Template Prep Kit 1.0 (Pacific Biosciences, Menlo Park, CA, USA) from gDNA size-selected to approximately 15 kb and sequenced on a PacBio Sequel system with the Sequel Sequencing Kit 2.1 (Pacific Biosciences). Short read libraries corresponding to 350 bp insert size were prepared using the TruSeq DNA PCR-Free Low Throughput Library Preparation Kit (Illumina, San Diego, CA, USA). Libraries were subjected to 150-bp paired-end sequencing on the HiSeq X Ten platform (Illumina). Hi-C library preparation, including cross-linking the DNA, *Mbo* I restriction enzyme digestion, end repair, DNA cyclization, and DNA purification, was performed by Frasergen Co. Ltd. (Wuhan, China). All DNA extractions, library preparations, and DNA sequencing was performed by BGI (Shenzhen, China).

For RNA-Seq, total RNA was extracted using TRIzol Reagent (Thermo Fisher Scientific, Carlsbad, CA, USA) and sequencing libraries were constructed using the TruSeq RNA v2 Kit (Illumina). The library was paired-end sequenced using a 150 cycle high output sequencing kit, on an Illumina NextSeq 500 instrument.

### Genome assembly

Quality control was performed for the raw Illumina short reads using BBTools suite v38.49 [50]: duplicates were removed using clumpy.sh; low-quality bases were trimmed using bbduk.sh, with a minimum Phred quality score of 20 for both read sides, a minimum read/Ns length of 15/5 bp, a maximum length poly-A/G/C tail of 10 bp. Genome properties was surveyed using GenomeScope v1.0.0 [51] with 1,000 and 10,000 as the maximum *k*-mer coverage cutoffs.

Raw PacBio long reads were assembled into contigs using Flye v2.5 [52] and Falcon v1.3.0 [53]. Both assemblies were self-polished with a round of the Flye polishing module “—polish-target”. To improve contig contiguity, Flye and Falcon assemblies were merged with two rounds of quickmerge [54] following USAGE 2 (https://github.com/mahulchak/quickmerge/wiki; last accessed March 1, 2021). We removed redundant heterozygous contigs with Purge Haplotigs v1.1.0 [55] with the percent cutoff for identification of each contig as a haplotig set as 50 (-a 50) t. Non-redundant assembly was further polished with Illumina short reads using two rounds of Pilon v1.22 [56]; input BAM mapping files were generated using Minimap2 v2.17 [57] and SAMtools v1.9 [58].

To perform the chromosome-level concatenation, we aligned Hi-C read pairs to the genome, removed duplicates, and extracted the Hi-C contacts using Juicer v1.6.2 [59]. Pseudo-chromosomes were generated using 3D-DNA v180922 [60] in combination with a review step in Juicebox Assembly Tools module within Juicebox [59]. To identify sex chromosomes Z and W, we mapped Illumina short reads using BWA (DOI: 10.1093/bioinformatics/btp698), then the average depth of coverage was calculated using SAMtools. Chromosomal nomenclature order and orientation were assigned by direct comparison with the *Bombyx mori* genome [61].

Sequences corresponding to contamination were removed by comparison with the nt and UniVec databases using HS-BLASTN v0.0.5 [62] and BLAST+ (i.e. blastn) v2.7.1 [63]. We assessed the assembly completeness using Benchmarking Universal Single-Copy Orthologs (BUSCO) [64] analyses against insect reference dataset (insect_odb9, n = 1,658). We also mapped PacBio long reads and Illumina short reads to the final genome assembly with Minimap2.

### Genome annotation

To identify repetitive DNA elements, we constructed a *de novo* species-specific repeat library using RepeatModeler v1.0.11 [65], and then combined it with Dfam_3.0 [66] and RepBase-20181026 databases [67] to generate a custom library. We then used RepeatMasker v4.0.9 [68] to mask repeats in the genome assembly with the combined custom library.

Protein-coding genes were predicted with the MAKER v2.31.10 pipeline [69] which can integrate *ab initio*, transcriptome- and protein homology-based evidence. For the *ab initio* gene predictions, we trained gene model parameters for Augustus v3.3 [70] and GeneMark-ET v4.38 [71] using BRAKER v2.1.0 [72], which incorporated RNA-seq data to accurately identify exon/intron boundaries. Input BAM alignment file for BRAKER was generated with HISAT2 v2.1.0 [73]. For the transcriptome-based evidence, a genome-guided method was applied to transcript assembly using StringTie v1.3.4 [74]. For the protein homology-based evidence, protein sequences of *Apis mellifera* (GCF_003254395.2), *B*. *mori* (GCF_000151625.1), *D*. *melanogaster* (GCF_000001215.4), *Tribolium castaneum* (GCF_000002335.3), *Helicoverpa armigera* (GCF_002156985.1) and *Spodoptera litura* (GCF_002706865.1) were downloaded directly from NCBI. Finally, repeat-masked genome and repeat annotations, assembled transcripts, protein sequences, and *ab initio* predicted gene models were passed to MAKER for automated gene structure predictions.

Gene functions were assigned to pro-coding gene models using Diamond v0.9.24 [75] against the UniProtKB (SwissProt + TrEMBL) database with the sensitive mode ‘—more-sensitive -e 1e-5’. We searched protein domains, Gene Ontology (GO) and Reactome pathway using InterProScan 5.41–78.0 [76] (-dp -f TSV, GFF3 -goterms -iprlookup -pa -t p -appl Pfam, Smart, Gene3D, Superfamily, CDD). We also annotated genes using eggNOG-mapper v2.0 [77] against the eggNOG v5.0 database [78] to obtain additional information of GO, Kyoto Encyclopedia of Genes and Genomes (KEGG) orthology (KO), KEGG pathways, enzyme codes (ECs), and clusters of orthologous groups (COGs). Circos figure was produced by Tbtools v1.0 [79].

### Bulked segregant analysis and mapping by sequencing

To generate EB-resistant individuals for BSA, a single female moth from WH-EB was mated with a male moth from JZ-S to generate F_1_ progeny. Because crossing over only occurs in males and EB resistance in WH-EB is incompletely dominant, a male F_1_ progeny was then backcrossed with a JZ-S female to generate backcross progeny for selection on EB (**Fig 2A**). A total of 336 individuals (3^rd^ instar larvae) from the backcross progeny were assayed on the diagnostic concentration of 0.01 μg/cm^2^ EB, of which 134 individuals survived and comprised the BC-Sel family. We collected and pooled a single leg from each of the 120 BC-Sel survivors (e.g., pool for BSA consisted of a total of 120 moth legs) as well as the whole bodies for each of their parents. Total gDNA was extracted using a Blood & Cell Culture DNA Mini Kit (Qiagen). DNA was fragmented by sonication to obtain ~350 bp fragments and used for library preparation using the NEBNext Ultra DNA Library Prep Kit (New England Biolabs, Ipswich, Massachusetts, USA). Libraries were sequenced on Illumina NovaSeq6000 PE-150bp sequencing system (Novogene Co. Ltd, Beijing, China). We sequenced 30x for each parent of the backcross family (male F_1_ hybrid and female JZ-S), and 240x for the pooled 120 moth legs from the BC-Sel family (2x per individual).

The raw short-read data were filtered for quality by Trimmomatic software (version 0.36) with the following parameters “HEADCROP: 20 SLIDINGWINDOW: 4: 15 MINLEN: 50” [80] and mapping to the reference sequence using MEM module in Burrows-Wheeler Aligner software [81]. The resulting alignment files were transformed to binary files and sorted by SAMtools software [58]. PCR duplicates were identified by GATK [82] and indexed using SAMtools [58]. The HaplotypeCaller module in GATK was used to call variants [82]. SNP variants in each VCF files were filtered for quality using GATK with the following parameters [QD < 2.0 || MQ < 40.0 || FS > 60.0 || SOR > 3.0 || MQRankSum < -12.5 || ReadPosRankSum < -8.0] [82].

Informative SNPs, which are heterozygous in the male F_1_ hybrid and homozygous in the female parent (JZ-S), were selected to indicate the SNP segregation in the backcross progeny. All informative SNPs were expected to segregate 1:3 (for autosomes) or 1:2 (for Z chromosome) in the untreated backcross progeny. The absolute values of deviation between the detected frequencies in the BC-Sel pool and the expected frequencies (0.25 or 0.33) were calculated for each informative SNP. The mean frequency deviation values obtained from a sliding window analysis (1 Mb window with 100 Kb step) were plotted across all 31 chromosomes of the beet armyworm genome. Sliding windows with fewer than 80 informative SNPs were discarded in order to reduce noise. The mean frequency deviation values for the SNPs on Z chromosome were multiplied by a correction factor 0.75 (0.25/0.33) to make them comparable with that on autosomes.

### Guide RNA design for CRISPR/Cas9

Oligonucleotide primers used to make *CYP9A40*, *CYP9A107*, *CYP9A186* and *CYP9A98* single guide RNA (sgRNA) templates are shown in **S4 Table**. Guide RNA was synthesized using the GeneArt Precision gRNA Synthesis kit (Thermo Fisher Scientific, Waltham, MA, USA) according to the manufacturer’s instructions. All PCR products were purified using a Qiagen PCR purification kit (Hilden, Germany) and used as templates for *in vitro* transcription. sgRNAs were purified and diluted in nuclease-free water to a concentration of ~2 μg μL^-1^ and stored in aliquots at -80°C.

### Egg collection and microinjection

Freshly laid WH-EB eggs (within 2 h) were collected from gauze using 1% (v/v) sodium hypochlorite solution and rinsed with distilled water. Eggs were affixed onto microscope slides using double-sided adhesive tape [33,34]. Individual eggs were injected with approximately 2 nL of a solution containing two sgRNAs and the Cas9 protein (Thermo Fisher Scientific, Waltham, MA, USA) using a Nanoject III (Drummond, Broomall, PA, USA). The final concentration of each sgRNA was ~ 400 ng/μL and the concentration of Cas9 protein was 100 ng/μL. All microinjections were completed within 2 h (eggs less than 4 h old). Injected eggs were incubated at 25°C and 65% RH for 3–4 days until they hatched.

### CRISPR/Cas9 knockout of *CYP9A* genes

To detect CRISPR/Cas9-induced mutations, primers (**S4 Table**) were designed and used to amplify gDNA flanking the CRISPR target sites. We obtained gDNA from individuals using the Multisource Genomic DNA Miniprep Kit (Axygen, New York, USA). PCR reactions contained 12.5 μL of 2x EasyTaq mix (TransGen, Beijing, China), 1 μL of each 10 μM sense and antisense primer (**S4 Table**), 1 μL gDNA, and 9.5 μL ddH_2_O. PCR was performed at 94°C for 3 min, 32 cycles of (94°C 20 s, 56°C 20 s, 72°C 2 min) and 72°C for 7 min. Five μL of each PCR reaction was separated on 1.25% agarose gels stained with ethidium bromide. DNA bands were gel-purified, TA-cloned into the pClone007 blunt vector (Tsingke BioTech, Beijing, China), and Sanger sequenced by Tsingke BioTech.

To create strains homozygous for the Cas9-induced mutations, 30–80 pairs of G_0_ moths were sib mated (**S5 Table**) and used to extract gDNA after laying eggs. PCR products flanking the two sgRNA target sites were amplified as indicated above to determine the genotypes and select lines to retain (**S5–S7 Figs**). At the 2^nd^ instar larval stage, gDNA from 10 G_1_ progeny was again extracted and used to detect the heritable mutations. Only progeny positive for the desired mutations were maintained (**S5 Table**). G_2_ individuals that were heterozygous for the desired deletion mutation were mass crossed to produce G_3_ (**S8 Fig**). Finally, homozygous G_3_ individuals harboring the deletion mutation were then selected and mass crossed to establish each homozygous strain (**S8 Fig**).

### Sequencing *CYP9A107*, *CYP9A27*, *CYP9A11*, *CYP9A186* and *CYP9A98* cDNA

To assess mutations in RNA, we PCR amplified full-length cDNAs corresponding to *CYP9A107*, *CYP9A27*, *CYP9A11*, *CYP9A186* and *CYP9A98*. cDNA was prepared from total RNA extracted from pools of five 3^rd^ instar larvae from WH-S and WH-EB. RNA was obtained using Trizol (Invitrogen, Carlsbad, CA, USA) according to the manufacturer’s instruction and treated with DNase I (TaKaRa, Shiga, Japan) to reduce genomic DNA contamination. First-strand cDNA synthesis was performed with 1 μg of total RNA using the Moloney Murine Leukemia Virus Reverse Transcriptase (M-MLV RT) cDNA synthesis kit (Promega, Madison, WI, USA).

Full-length cDNAs corresponding to *CYP9A107*, *CYP9A27*, *CYP9A11*, *CYP9A186* and *CYP9A98* were PCR amplified using specific primers (**S6 Table**) with reaction mixtures containing 10 μL 5x Q5 reaction buffer (New England Biolabs, Ipswich, MA, USA), 0.5 μL dNTPs (10 μM), 1.25 μL each of the sense and antisense primers (10 μM), 2 μL cDNA, 0.25 μL Q5 High-Fidelity DNA polymerase (New England Biolabs), and 9.75 μL ddH_2_O in a final volume of 25 μL. PCR conditions were 98°C 30 s, 35 cycles of 98°C 10 s, 60°C 20 s, 72°C for 2 min, and a final extension at 72°C for 2 min. PCR products were separated on 1.25% agarose gels in Tris-acetate-EDTA buffer and stained with ethidium bromide. PCR products of expected sizes were gel-purified with an AxyPrep Gel extraction kit (Axygen Biosciences, Union City, CA, USA), and then cloned into the pClone007 vector (Tsingke BioTech). *Escherichia coli Trans1*-T1 (Transgen Biotech) were transformed and inserts were sequenced by Tsingke BioTech. A total of 5 clones were sequenced for each cDNA for both WH-S and WH-EB.

### Quantitative real-time PCR analysis of *CYP9A107*, *CYP9A27*, *CYP9A11*, *CYP9A186* and *CYP9A98*

For RT-qPCR analysis of *CYP9A107*, *CYP9A27*, *CYP9A11*, *CYP9A186* and *CYP9A98*, we isolated total RNA from the midgut and fat bodies of individual WH-S and WH-EB 5^th^ instars. First-strand cDNA was synthesized using 2 μg of total RNA using PrimeScript Reverse Transcriptase (TaKaRa, Dalian, China) according to the manufacturer’s protocol. The reaction cycling conditions for the five *CYP9A*s are identical to those used by Shi et al. [83]. Four biological replicates were done for each gene. qPCR results were analyzed using the 2^-△△^Ct method [84]. Data were normalized to the geometric mean of two housekeeping genes (*β-actin* and *GADPH*). Significant differences in transcript abundance were determined using the Student’s *t*-test (*α* = 0.05) using SPSS 20.0 software (SPSS Inc., Chicago, IL, USA).

### Chemicals and bioassays

Emamectin Benzoate (EB) (~93.4%B1a, CAS: 155569-91-8, 98%), 7-benzyloxy-4-trifluoromethyl coumarin (BFC) and reduced nicotinamide adenine dinucleotide phosphate (NADPH) were purchased from Sigma-Aldrich company (St. Louis, Missouri, USA). Abamectin (B1a, CAS: 65195-55-3, 97%) was purchased from Toronto Research Chemicals (North York, Canada). Solvents for LC-MS (ammonium acetate, formic acid and acetonitrile) were obtained from Fisher Scientific (Pittsburgh, Pennsylvania, USA).

A series of gradient concentrations of insecticides diluted from stock solution with 0.1% (w/v) Triton X-100 water were prepared. In each well of the 24-well plate, a liquid artificial diet (~120 μL) was supplied. One hundred microliters of the insecticide solution was added to the dietary surface of every single well after the diet cooled and solidified. A third-instar larva was placed in each well. For each concentration, 24 larvae were treated. Triton X-100 (0.1%, w/v) was employed to process the control group. Two days later, larvae were checked and considered dead if they did not move after gentle prodding. The data were analyzed using DPS 7.05 software (Zhejiang University, Hangzhou, China). When the 95% fiduciary limits of the LC_50_ values did not overlap, the LC_50_ values were considered to vary significantly.

### Heterologous expression of CYP9A186

Full-length cDNAs corresponding to the ORFs of wild-type *CYP9A186* (GenBank accession no. MN179467) and mutant *CYP9A186* (CYP9A186-F116V) (GenBank accession no. MN179472) were subcloned into pFastBac1 (Invitrogen) from WH-S and WH-EB, respectively. Controls included the beet armyworm cytochrome P450 reductase (SeCPR, GenBank accession no. MN179471) subcloned from WH-S and cells infected with virus containing empty pFastBac1 vector (non-insertion negative control). Heterologous protein expression was carried out using the Bac-to-Bac system (Invitrogen) as previously described [83]. Briefly, Hi5 cells were maintained at 27°C with 130 rpm mixing in Express Five serum-free medium (Invitrogen). P3 virus was added when Hi5 cell density reached 2–3×10^6^ cells mL^-1^. Cells were co-infected with recombinant virus (either CYP9A186, CYP9A186-F116V, or non-insertion control) and SeCPR with multiplicity of infection (MOI) of 2 and 0.2, respectively. Ferric citrate (0.1 mM) and 5’-aminolevulinic acid hydrochloride (0.1 mM) were added to the medium along with virus and 24 h post infection. Microsomes were prepared by differential centrifugation. Total microsomal protein content was determined using the Bradford method [85] and the amount of recombinant P450 was quantified using the reduced CO-difference spectral assay [86].

### *In vitro* metabolism of model substrates and insecticides

To observe the metabolism exhibited by CYP9A186 and CYP9A186-F116V, *in vitro* enzyme assays were performed on microsomal fractions containing recombinant P450/SeCPR (or non-insertion control/SeCPR). All assays were performed in 0.1 M potassium phosphate buffer (pH 7.4) with various substrates in a total reaction volume of 200 μL.

To test for the presence of active recombinant cytochrome P450s (indicating correct protein folding), 25 mM 7-benzyloxy-4-trifluoromethyl coumarin (BFC) (dissolved in DMSO) was added to 20 pmol aliquots of each microsomal protein sample in black, flat-bottom 96-well plates for 5 min at 30°C. Reactions were initiated by adding 10 μL of 10 mM NADPH. The formation of metabolized product was monitored using a Spectra Max M5 plate reader (Molecular Devices) as previously described [83]. Controls included samples without NADPH and microsomal samples from “no insert”-infected cells (non-insertion control). Specific activities were corrected by subtracting the background (activity of non-insertion control) and expressed as pmol product per minute per pmol P450.

To measure the metabolism of EB and abamectin directly, 10 pmol aliquots of each microsomal protein sample was incubated with an NADPH regenerating system (1.3 mM NADP^+^, 3.3 mM glucose-6-phosphate, 3.3 mM MgCl_2_ and 0.4 U mL^-1^ glucose-6-phosphate dehydrogenase) and 10 μM of either insecticide (2 μL dissolved in DMSO). Reactions were pre-incubated for 5 min at 30°C before adding insecticide substrate. Reactions were carried out in an orbital shaking incubator at 30°C at 1,200 rpm. Reactions were stopped by adding 200 μL acetonitrile and 600 μL dilution buffer (50% potassium phosphate buffer and 50% acetonitrile). Each reaction was further incubated/shaken for an additional 20 min. Samples were centrifuged at at 18,000 × g for 10 min and supernatants (800 μL) were transferred to HPLC vials and analyzed immediately. Control samples (e.g., without NADPH and non-insertion negative controls) containing equal amount of total protein were subsequently tested. The appearance of degraded insecticides indicates metabolic enzymatic activity. Specific activities were corrected by subtracting the background (activity of non-insertion negative control) and expressed as pmol substrate/min/pmol P450.

### Identification of insecticide metabolites by UPLC-MS/MS

The degradation of EB and abamectin was monitored and quantified by UPLC-MS/MS. Samples (0.5 μL each) were separated by Acquity BEH C8 column (2.1 × 50 mm, 1.7 μm particle size, Waters, Milford, Massachusetts) using a Waters Acquity UPLC system (Waters ACQUITY UPLC I-Class) and eluted with a gradient of mobile phase consisting of A: 10 mM ammonium acetate [+0.1% (*v*/*v*) formic acid] and B: acetonitrile, with a constant flow rate of 0.3 mL min^-1^. The gradient elution conditions were as follows: 0 min A: B 50: 50; 0.3 min A: B 50: 50; 2 min A: B 5:95; 2.5 min A: B 5: 95; 2.6 min A: B 0:100; 3 min A: B 0: 100; 3.1 min acetonitrile: Water 50: 50; 5 min acetonitrile: Water 50: 50. Samples separated by UPLC were directly analyzed using a tandem triple quadrupole mass-spectrometer (Waters Xevo TQ-S micro, Waters, Milford, Massachusetts) and run in positive ESI mode with multireaction monitoring (MRM) (**S7 Table**).

For identification of the EB and abamectin metabolites, samples were separated using a Shimadzu LC system (LC20ADXR with Agilent Poroshell 120 EC-C18 column 2.1 mm × 50 mm, 2.7 μm) and the same mobile phases and gradient as outlined above. The partitioned samples were then analyzed by high-resolution mass spectroscopy (Triple Tof 5600+, AB Sciex, USA) under +ESI mode with collision energy at 50V.

## Supporting information

S1 Fig Schematic diagram of the sgRNA targeting sites and the CRISPR/Cas9 CYP9A186 mutagenesis. (**A**) Schematic diagram of the sgRNA-targeting sites. The gray line indicates the genome locus of *CYP9A186* and the boxes represent the exons of *CYP9A186*. The sgRNA-targeting site was located on the sense strand of exon 1. The sgRNA-targeting sequence is shown in purple, and the protospacer adjacent motif (PAM) sequence is in red. (**B**) Representative chromatograms of PCR-product sequencing in G_1_ individuals and the CYP9A186 knockout strain (dA186) showing presence of 4-bp indel mutation. (**C**) Diagram detailing crossing scheme used to obtain the homozygous CYP9A186 knockout strain (+, wild-type; -, mutant).
(TIFF)

S2 Fig Reduced CO difference spectra of recombinant CYP9A186 F116V mutant, wild type and non-insertion control. (TIFF)

S3 Fig Formation of hydroxy- and O-desmethy-metabolites in *in vitro* metabolism (MRM spectra). Metabolism of emamectin benzoate: **A**, samples with CYP9A186-F116V; **B**, samples with CYP9A186wt; **C**, samples with non-insertion control (CK). Metabolism of abamectin: **D**, samples with CYP9A186-F116V; **E**, samples with CYP9A186wt; **F**, samples with non-insertion control (CK). MRM spectra of parent compound (emamectin benzoate and abamectin), hydroxy-metabolite (-OH) and O-desmethyl-metabolite (-O-CH_3_) were shown in the top, middle, and bottom respectively within each block. Red arrows indicate metabolites.
(TIFF)

S4 Fig ESI-TOF high resolution MS spectrum of metabolites resulting from incubations of emamectin benzoate and abamectin with recombinantly expressed CYP9A186-F116V. (**A**) Mass spectra of the metabolite hydroxyl-emamectin benzoate (HO-groups correspond to either 24’ or 26’ position). (**B**) Mass spectra of the metabolite O-desmethyl-emamectin benzoate. (**C**) Mass spectra of the metabolite hydroxyl-abamectin (HO-groups correspond to either 24’ or 26’ position). (**D**) Mass spectra of the metabolite O-desmethyl-abamectin.
(TIFF)

S5 Fig CRISPR/Cas9-based knockout of the *CYP9A* cluster (dA40-A98) in the beet armyworm WH-EB strain. (**A**) sgRNA targeting site of *CYP9A40* and *CYP9A98* genes and the two primer pairs for allele-specific PCR detection. Target sequences and protospacer adjacent motifs (PAMs) are shown in light blue and in red, respectively. The positions of the two sgRNAs (sgRNA-A40 and sgRNA-A98) and a representative chromatogram of direct sequencing of PCR products of individuals from the dA40-A98 strain with the primer pair A40F/A98R are shown. (**B**) Genotyping of individual *S*. *exigua* for deletion of the *CYP9A* cluster according to banding patterns of the PCR products amplified with a set of five primer pairs. M, 2000 bp MW Marker; Lane 1, A40F/A98R; Lane 2, A40F/A40R; Lane 3, A9F/A9R; Lane 4, A186F1/A186R1; and Lane 5, A98F/A98R.
(TIFF)

S6 Fig CRISPR/Cas9-based knockout of the *CYP9A* cluster (dA40-A107) in the beet armyworm WH-EB strain. (**A**) sgRNA targeting site of *CYP9A40* and *CYP9A107* genes and the two primer pairs for allele-specific PCR detection. Target sequences and protospacer adjacent motifs (PAMs) are shown in light blue and in red, respectively. The positions of the two sgRNAs (sgRNA-A40 and sgRNA-A107) and a representative chromatogram of direct sequencing of PCR products of individuals from the dA40-A107 strain with the primer pair A40F/A107R are shown. (**B**) Genotyping of individual *S*. *exigua* for deletion from *CYP9A40* to *CYP9A107* according to banding patterns of the PCR products amplified with a set of five primer pairs. M, 2000 bp MW Marker; Lane 1, A40F/A107R; Lane 2, A40F/A40R; Lane 3, A107F/A107R; Lane 4, A9F/A9R; and Lane 5, A186F2/A186R2.
(TIFF)

S7 Fig CRISPR/Cas9-based knockout of the *CYP9A* cluster (dA107-A98) in the beet armyworm WH-EB strain. (**A**) sgRNA targeting site of *CYP9A107* and *CYP9A98* genes and the two primer pairs for allele-specific PCR detection. Target sequences and protospacer adjacent motifs (PAMs) are shown in light blue and in red, respectively. The positions of the two sgRNAs (sgRNA-A107 and sgRNA-A98) and a representative chromatogram of direct sequencing of PCR products of individuals from the dA107-A98 strain with the primer pair A107F/A98R are shown. (**B**) Genotyping of individual *S*. *exigua* for deletion from *CYP9A107* to *CYP9A98* according to banding patterns of the PCR products amplified with a set of five primer pairs. M, 2000 bp MW Marker; Lane 1, A107F/A98R; Lane 2, A107F/A107R; Lane 3, A186F2/A186R2; and Lane 4, A98F/A98R.
(TIFF)

S8 Fig Diagram detailing crossing scheme used to obtain homozygous knockout strains of *Spodoptera exigua*. (TIFF)

S1 Table Genome survey for *Spodoptera exigua*. (DOCX)

S2 Table Summary of each assembly version for *Spodoptera exigua*. (DOCX)

S3 Table Repeat annotation in *Spodoptera exigua*. (DOCX)

S4 Table Primers for template DNA for in vitro transcription of sgRNAs and amplifying genomic DNA fragment of CYP9A genes. (DOCX)

S5 Table Mutagenesis induced by gRNA/Cas9. (DOCX)

S6 Table Primers for amplifying *SeCYP9A* subfamily genes and analysis *SeCYP9A* subfamily genes expression. (DOCX)

S7 Table Analysis parameters for substrates and metabolites detection. (DOCX)
