# Supplementary figures and images for: Genome mapping coupled with CRISPR gene editing reveals a P450 gene confers avermectin resistance in the beet armyworm
Source: PLoS Genet. 2021 Jul 12;17(7):e1009680. doi: 10.1371/journal.pgen.1009680 (PMC8297932; doi:10.1371/journal.pgen.1009680)

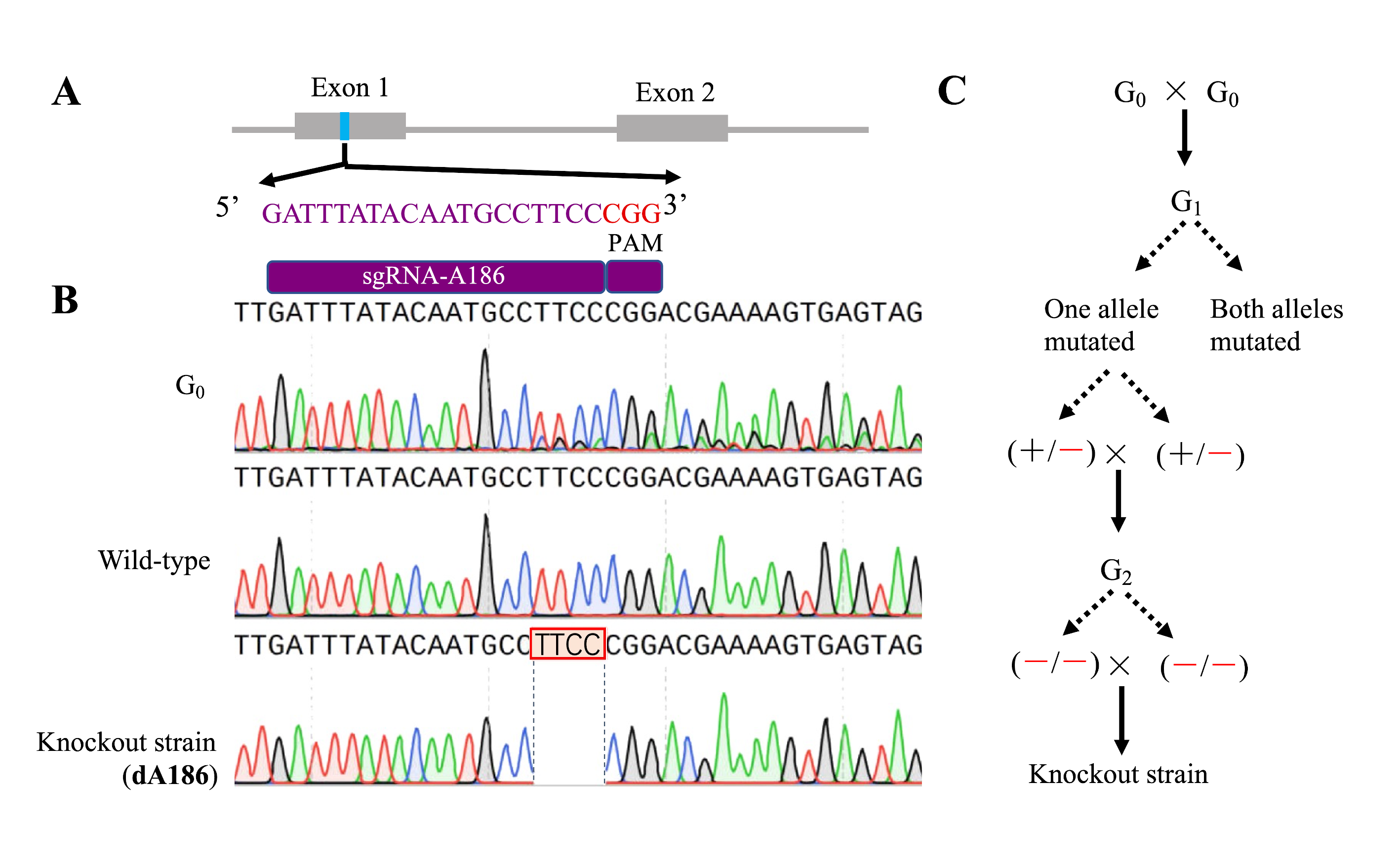

Supplement: S1 Fig — (A) Schematic diagram of the sgRNA-targeting sites. The gray line indicates the genome locus of CYP9A186 and the boxes represent the exons of CYP9A186. The sgRNA-targeting site was located on the sense strand of exon 1. The sgRNA-targeting sequence is shown in purple, and the protospacer adjacent motif (PAM) sequence is in red. (B) Representative chromatograms of PCR-product sequencing in G1 individuals and the CYP9A186 knockout strain (dA186) showing presence of 4-bp indel mutation. (C) Diagram detailing crossing scheme used to obtain the homozygous CYP9A186 knockout strain (+, wild-type; -, mutant). (TIFF) [file pgen.1009680.s001.tiff]

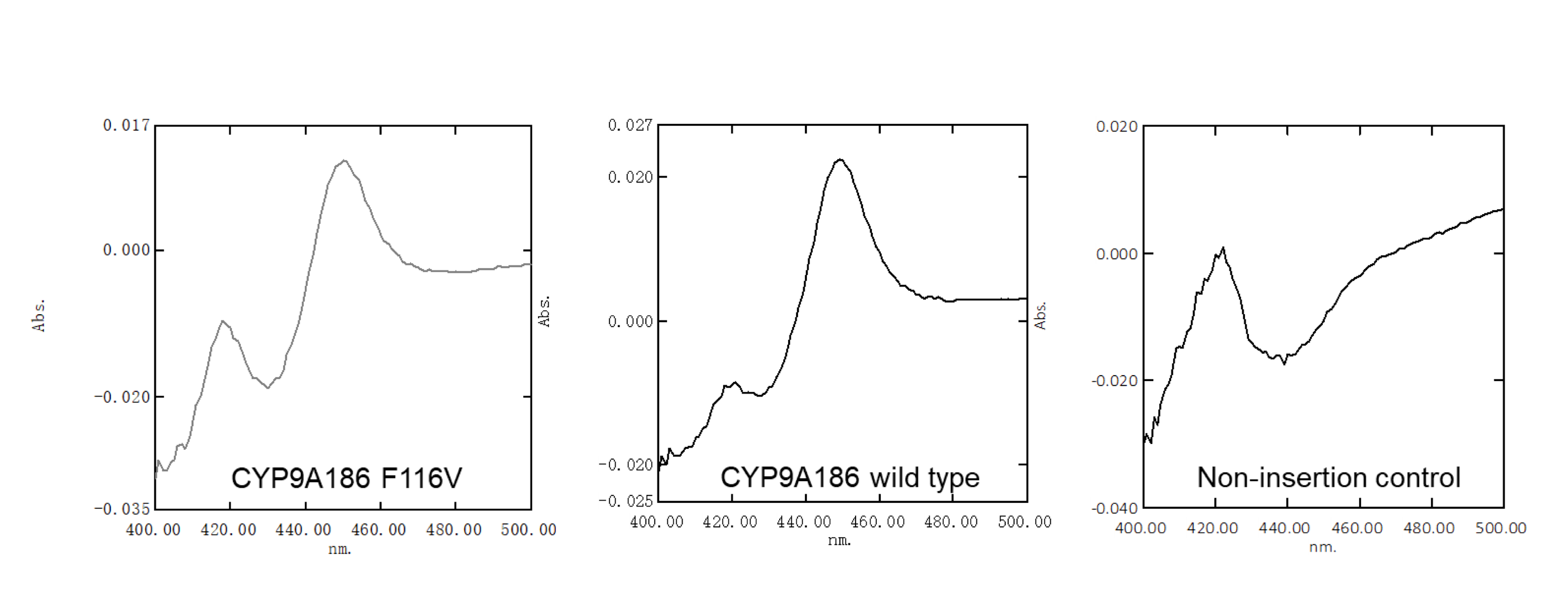

Supplement: S2 Fig — (TIFF) [file pgen.1009680.s002.tiff]

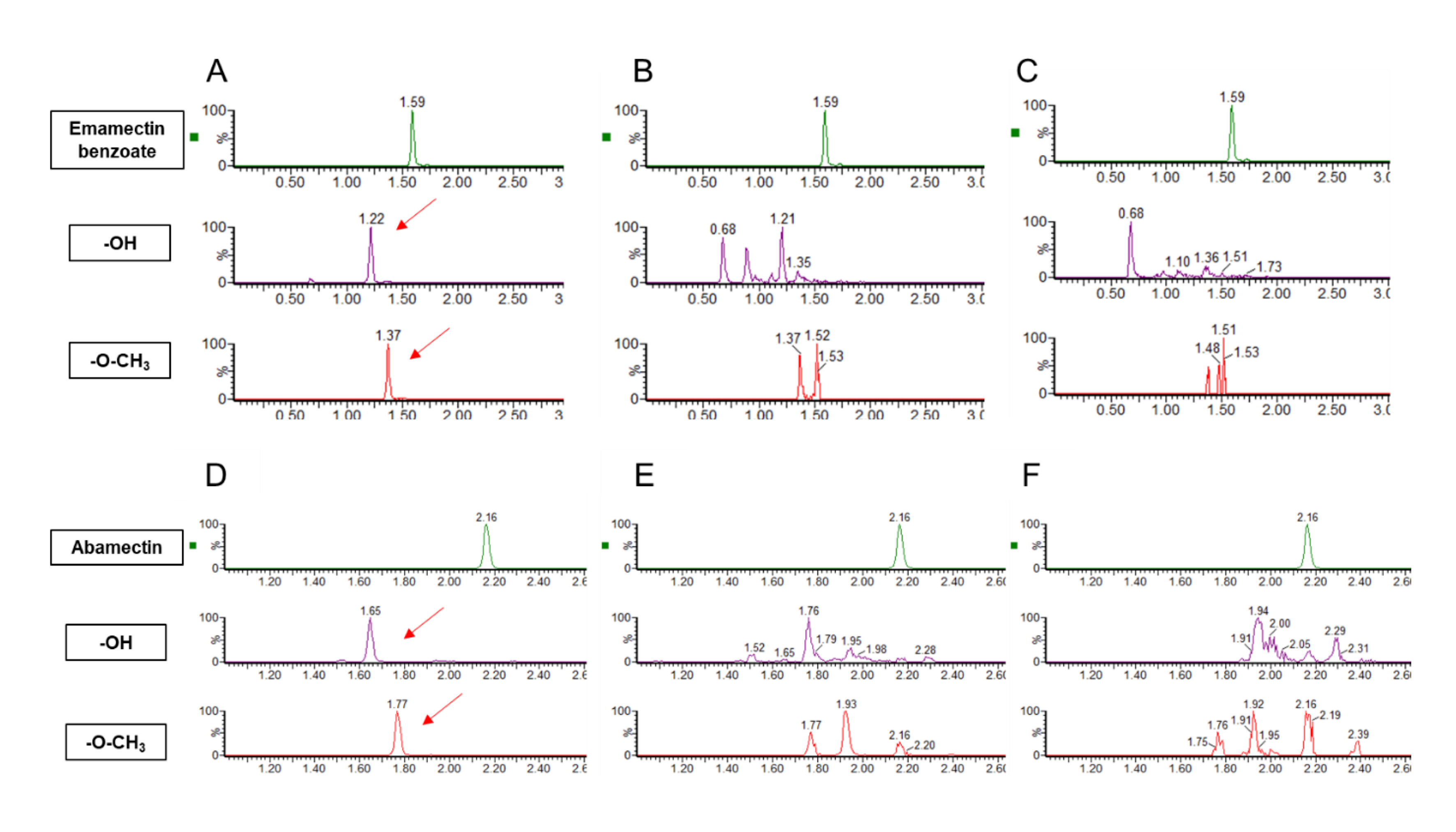

Supplement: S3 Fig — Metabolism of emamectin benzoate: A, samples with CYP9A186-F116V; B, samples with CYP9A186wt; C, samples with non-insertion control (CK). Metabolism of abamectin: D, samples with CYP9A186-F116V; E, samples with CYP9A186wt; F, samples with non-insertion control (CK). MRM spectra of parent compound (emamectin benzoate and abamectin), hydroxy-metabolite (-OH) and O-desmethyl-metabolite (-O-CH3) were shown in the top, middle, and bottom respectively within each block. Red arrows indicate metabolites. (TIFF) [file pgen.1009680.s003.tiff]

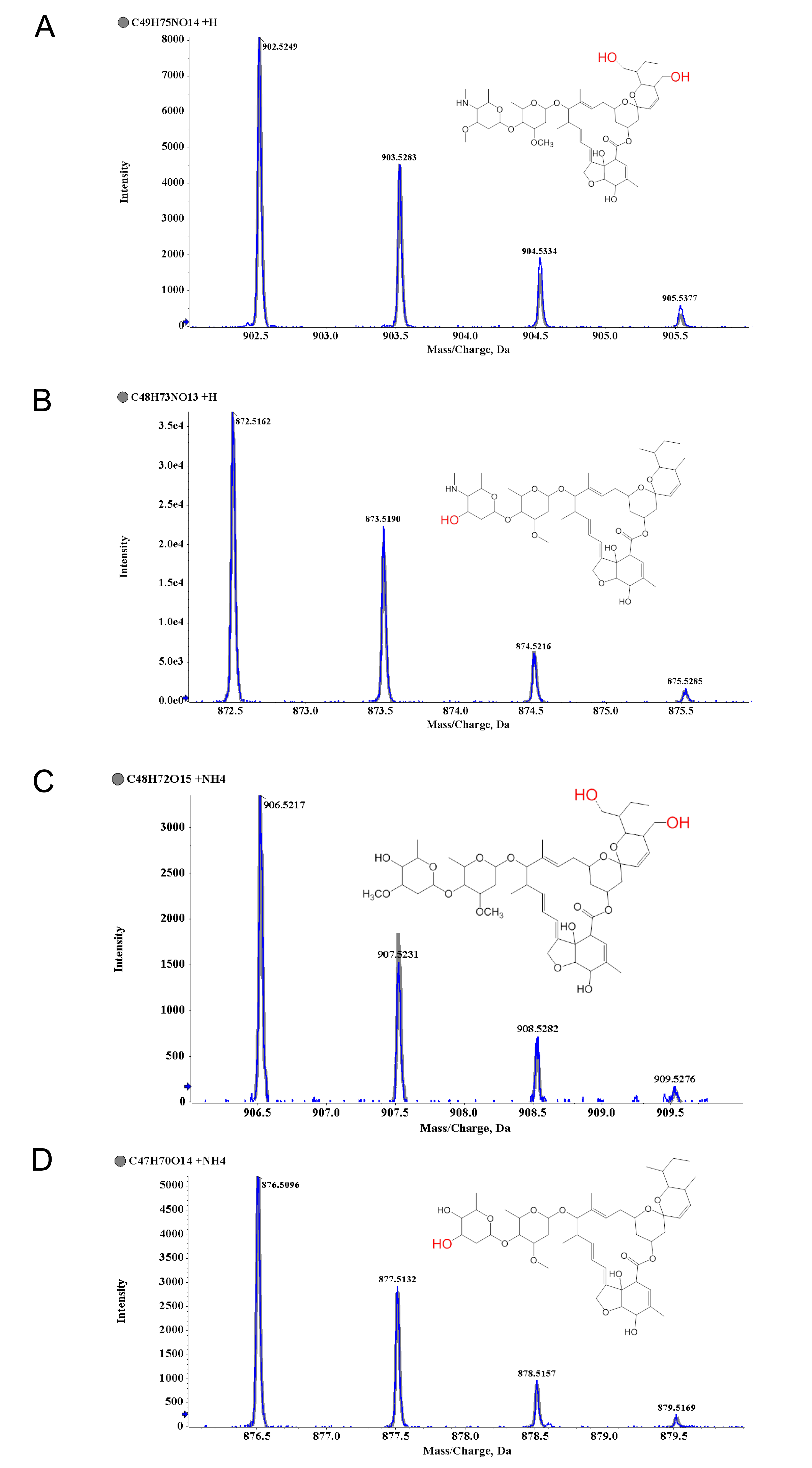

Supplement: S4 Fig — (A) Mass spectra of the metabolite hydroxyl-emamectin benzoate (HO-groups correspond to either 24’ or 26’ position). (B) Mass spectra of the metabolite O-desmethyl-emamectin benzoate. (C) Mass spectra of the metabolite hydroxyl-abamectin (HO-groups correspond to either 24’ or 26’ position). (D) Mass spectra of the metabolite O-desmethyl-abamectin. (TIFF) [file pgen.1009680.s004.tiff]

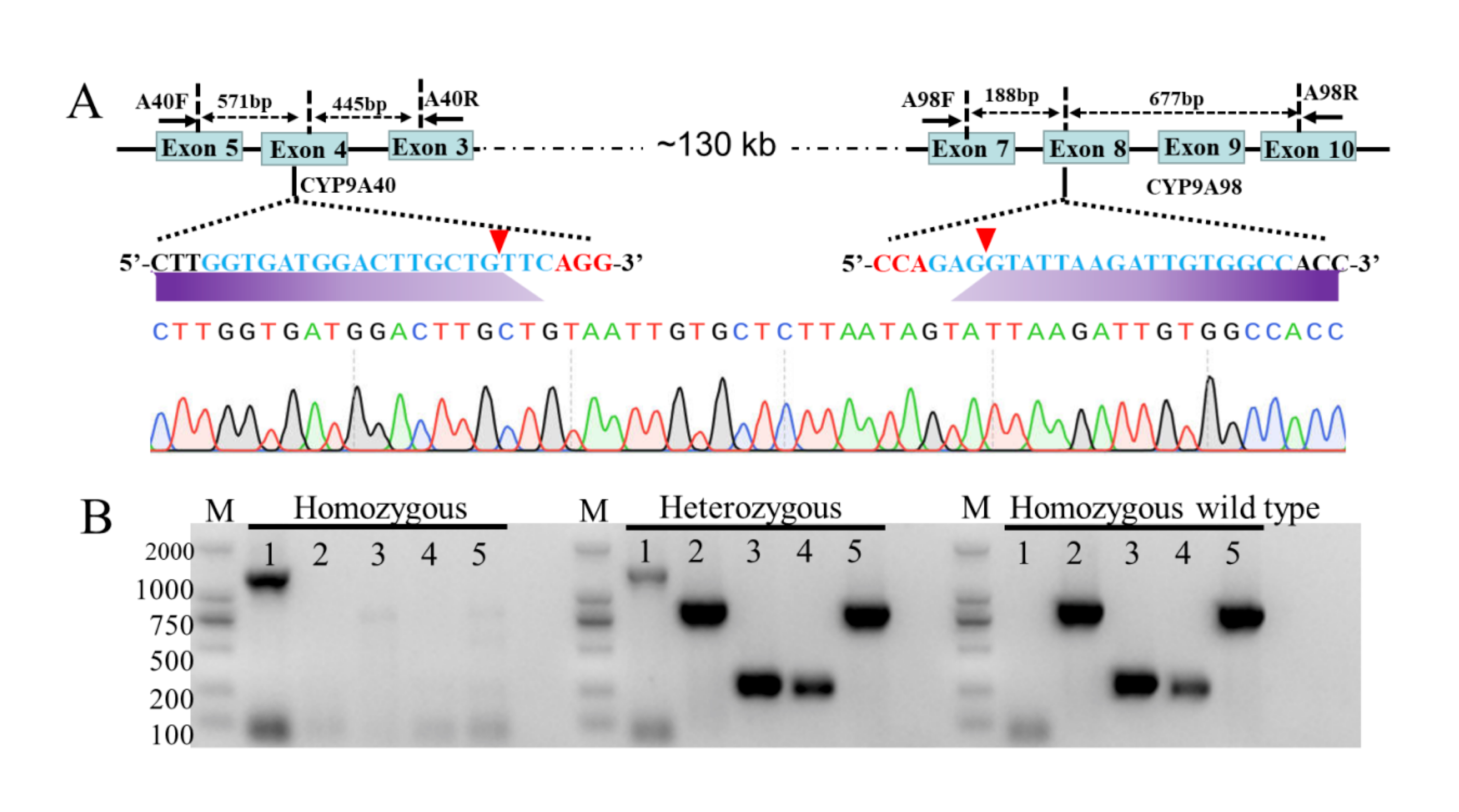

Supplement: S5 Fig — (A) sgRNA targeting site of CYP9A40 and CYP9A98 genes and the two primer pairs for allele-specific PCR detection. Target sequences and protospacer adjacent motifs (PAMs) are shown in light blue and in red, respectively. The positions of the two sgRNAs (sgRNA-A40 and sgRNA-A98) and a representative chromatogram of direct sequencing of PCR products of individuals from the dA40-A98 strain with the primer pair A40F/A98R are shown. (B) Genotyping of individual S. exigua for deletion of the CYP9A cluster according to banding patterns of the PCR products amplified with a set of five primer pairs. M, 2000 bp MW Marker; Lane 1, A40F/A98R; Lane 2, A40F/A40R; Lane 3, A9F/A9R; Lane 4, A186F1/A186R1; and Lane 5, A98F/A98R. (TIFF) [file pgen.1009680.s005.tiff]

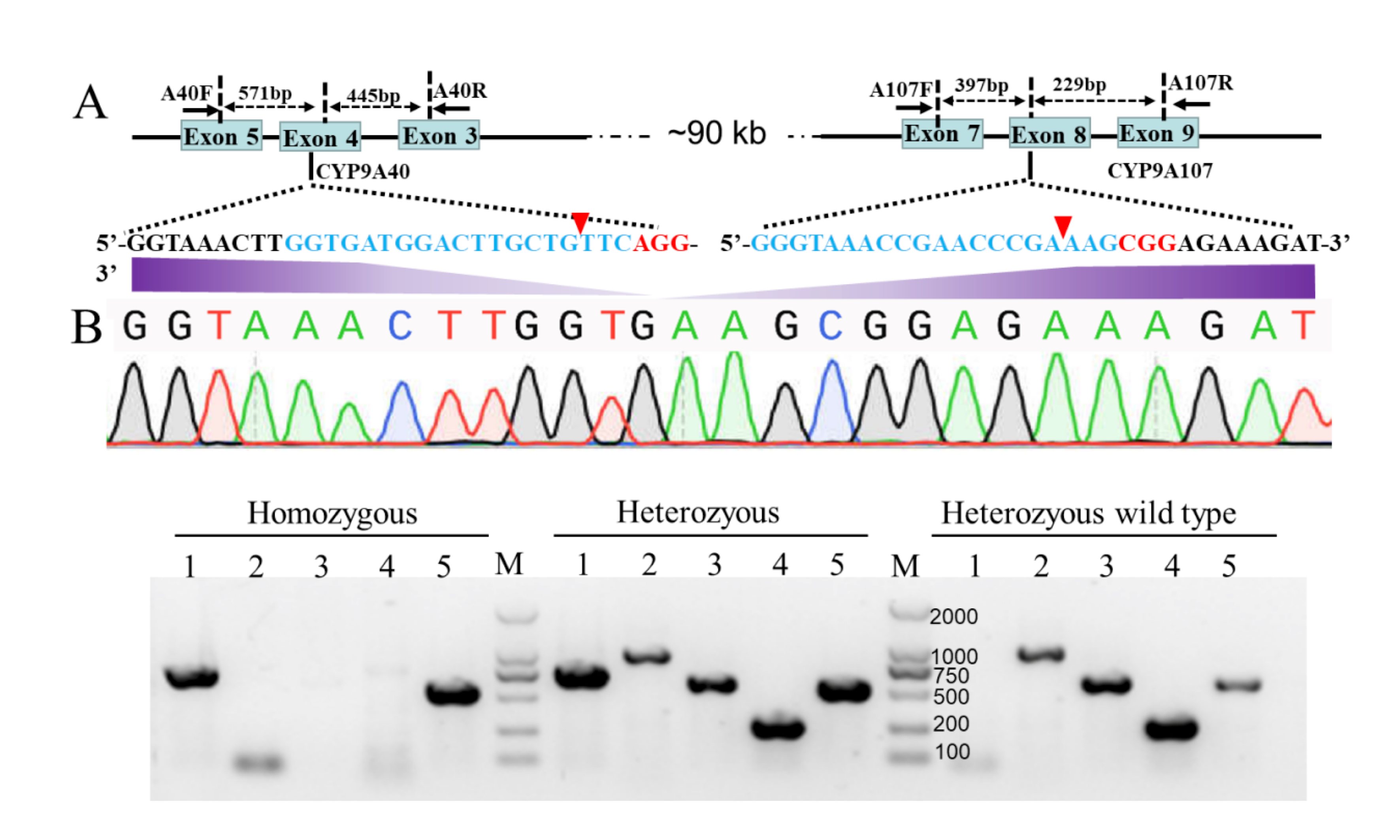

Supplement: S6 Fig — (A) sgRNA targeting site of CYP9A40 and CYP9A107 genes and the two primer pairs for allele-specific PCR detection. Target sequences and protospacer adjacent motifs (PAMs) are shown in light blue and in red, respectively. The positions of the two sgRNAs (sgRNA-A40 and sgRNA-A107) and a representative chromatogram of direct sequencing of PCR products of individuals from the dA40-A107 strain with the primer pair A40F/A107R are shown. (B) Genotyping of individual S. exigua for deletion from CYP9A40 to CYP9A107 according to banding patterns of the PCR products amplified with a set of five primer pairs. M, 2000 bp MW Marker; Lane 1, A40F/A107R; Lane 2, A40F/A40R; Lane 3, A107F/A107R; Lane 4, A9F/A9R; and Lane 5, A186F2/A186R2. (TIFF) [file pgen.1009680.s006.tiff]

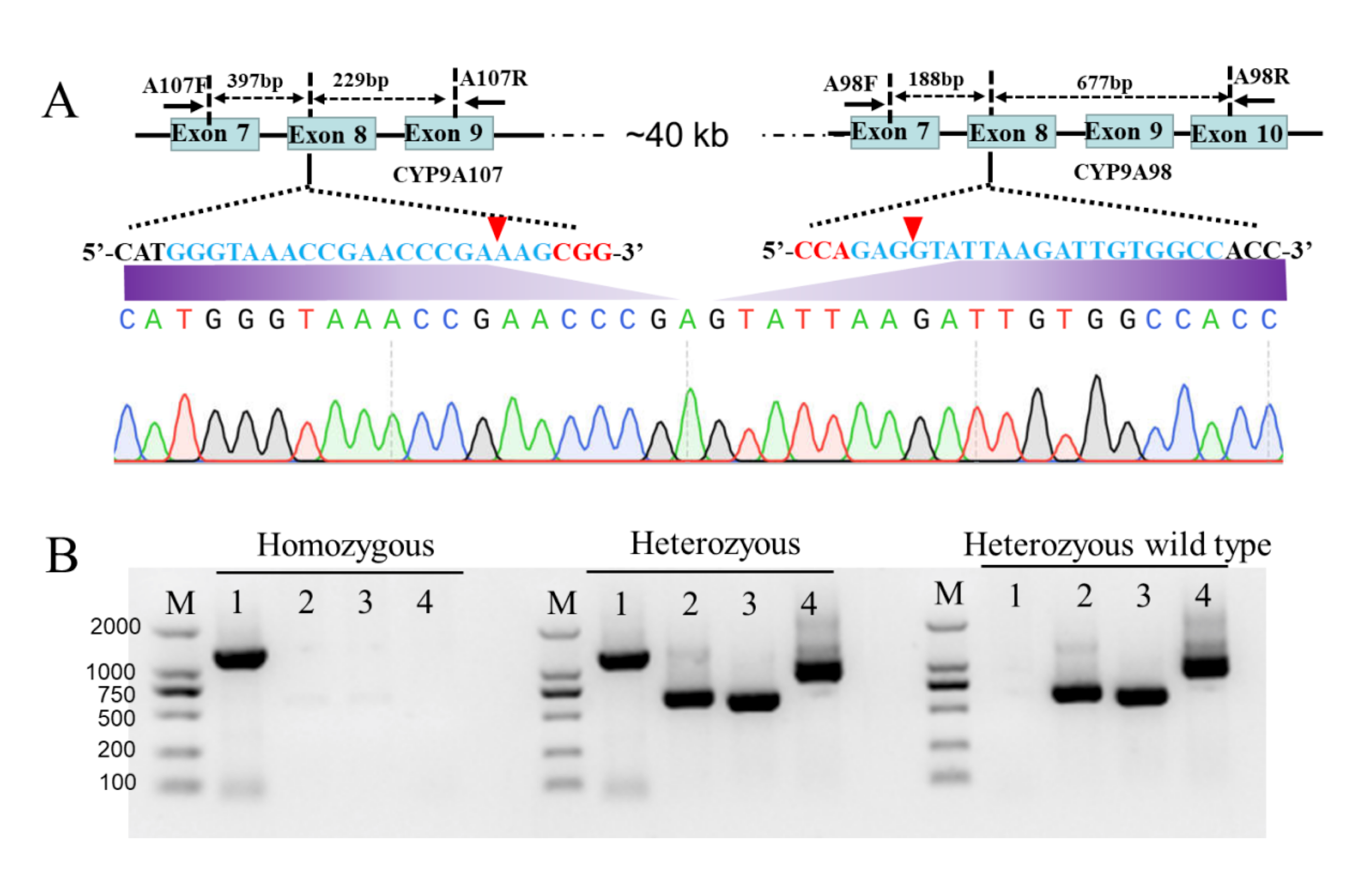

Supplement: S7 Fig — (A) sgRNA targeting site of CYP9A107 and CYP9A98 genes and the two primer pairs for allele-specific PCR detection. Target sequences and protospacer adjacent motifs (PAMs) are shown in light blue and in red, respectively. The positions of the two sgRNAs (sgRNA-A107 and sgRNA-A98) and a representative chromatogram of direct sequencing of PCR products of individuals from the dA107-A98 strain with the primer pair A107F/A98R are shown. (B) Genotyping of individual S. exigua for deletion from CYP9A107 to CYP9A98 according to banding patterns of the PCR products amplified with a set of five primer pairs. M, 2000 bp MW Marker; Lane 1, A107F/A98R; Lane 2, A107F/A107R; Lane 3, A186F2/A186R2; and Lane 4, A98F/A98R. (TIFF) [file pgen.1009680.s007.tiff]

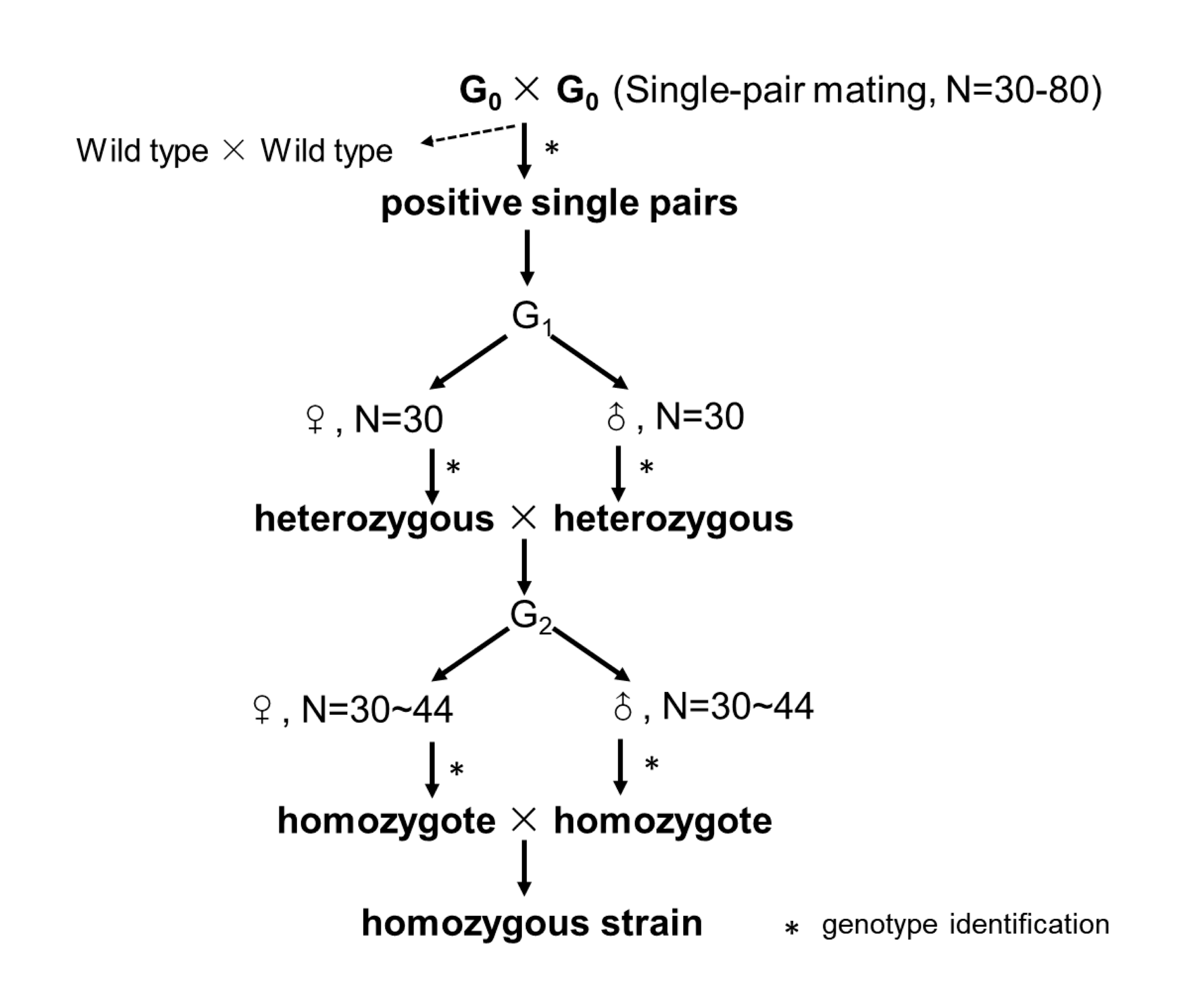

Supplement: S8 Fig — (TIFF) [file pgen.1009680.s008.tiff]
