# Supplementary material for: Genome mapping coupled with CRISPR gene editing reveals a P450 gene confers avermectin resistance in the beet armyworm
Source: PLoS Genet. 2021 Jul 12;17(7):e1009680. doi: 10.1371/journal.pgen.1009680 (PMC8297932; doi:10.1371/journal.pgen.1009680)
Supplement: S1 Table — (DOCX) [file pgen.1009680.s009.docx]

**S1 Table. Genome survey for *Spodoptera exigua*.**

| K-mer | Max k-mer  coverage | Heterozygosity (%) | | Repeat Length (Mb) | | Unique Length (Mb) | | Genome size (Mb) | |
| --- | --- | --- | --- | --- | --- | --- | --- | --- | --- |
|  |  | Min | Max | Min | Max | Min | Max | Min | Max |
| 21 | 1000 | 0.585 | 0.594 | 64.96 | 65.07 | 343.62 | 344.16 | 408.58 | 409.22 |
| 21 | 10000 | 0.588 | 0.591 | 104.88 | 104.93 | 343.80 | 343.97 | 448.68 | 448.90 |
