# Supplementary material for: Genome mapping coupled with CRISPR gene editing reveals a P450 gene confers avermectin resistance in the beet armyworm
Source: PLoS Genet. 2021 Jul 12;17(7):e1009680. doi: 10.1371/journal.pgen.1009680 (PMC8297932; doi:10.1371/journal.pgen.1009680)
Supplement: S2 Table — (DOCX) [file pgen.1009680.s010.docx]

**S2 Table. Summary of each assembly version for *Spodoptera exigua.***

| Assembly | Total length (Mb) | No. scaffolds | N50 length (kb) | Longest scaffold (Mb) | GC (%) | BUSCO (n=1,658) (%) | | | |
| --- | --- | --- | --- | --- | --- | --- | --- | --- | --- |
|  |  |  |  |  |  | C | D | F | M |
| Flye | 544.64 | 2,339 | 810 | 14.84 | 36.81 | 98.6 | 10.9 | 0.3 | 1.1 |
| Falcon | 559.54 | 1,061 | 1,600 | 14.32 | 36.71 | 98.4 | 16.6 | 0.2 | 1.4 |
| quickmerge | 561.68 | 934 | 3,474 | 22.01 | 36.71 | 98.1 | 16.9 | 0.4 | 1.5 |
| purge_haplotigs | 447.05 | 266 | 5,529 | 22.01 | 36.7 | 97.8 | 2.4 | 0.3 | 1.9 |
| Pilon | 446.52 | 266 | 5,526 | 22.01 | 36.67 | 98 | 2.2 | 0.2 | 1.8 |
| Final genome assembly | 446.80 | 301 | 14,363 | 19.74 | 36.67 | 97.9 | 2.1 | 0.2 | 1.9 |

C, complete BUSCOs; D, complete and duplicated BUSCOs; F, fragmented BUSCOs; M, missing BUSCOs.
