# Supplementary material for: Genome mapping coupled with CRISPR gene editing reveals a P450 gene confers avermectin resistance in the beet armyworm
Source: PLoS Genet. 2021 Jul 12;17(7):e1009680. doi: 10.1371/journal.pgen.1009680 (PMC8297932; doi:10.1371/journal.pgen.1009680)
Supplement: S3 Table — (DOCX) [file pgen.1009680.s011.docx]

**S3 Table. Repeat annotation in *Spodoptera exigua.***

| Class | Copies | Length (bp) | Percentage of the genome (bp) |
| --- | --- | --- | --- |
| ARTEFACT | 1 | 59 | 0.00% |
| DNA |  |  |  |
| Academ-1 | 604 | 121,008 | 0.03% |
| Academ-2 | 37 | 4,938 | 0.00% |
| Academ-H | 4 | 128 | 0.00% |
| CMC-Chapaev | 65 | 3,511 | 0.00% |
| CMC-Chapaev-3 | 131 | 46,889 | 0.01% |
| CMC-EnSpm | 1,139 | 102,021 | 0.02% |
| CMC-Transib | 178 | 66,522 | 0.01% |
| Crypton | 22 | 847 | 0.00% |
| Crypton-A | 6 | 266 | 0.00% |
| Crypton-C | 5 | 553 | 0.00% |
| Crypton-F | 8 | 279 | 0.00% |
| Crypton-H | 84 | 3,103 | 0.00% |
| Crypton-I | 4 | 273 | 0.00% |
| Crypton-R | 1 | 39 | 0.00% |
| Crypton-S | 10 | 898 | 0.00% |
| Crypton-V | 58 | 3,007 | 0.00% |
| Crypton-X | 1 | 37 | 0.00% |
| Dada | 96 | 6,408 | 0.00% |
| Ginger-1 | 75 | 4,587 | 0.00% |
| Ginger-2 | 140 | 24,867 | 0.01% |
| IS3EU | 69 | 3,904 | 0.00% |
| Kolobok | 6 | 463 | 0.00% |
| Kolobok-E | 67 | 19,949 | 0.00% |
| Kolobok-H | 4 | 194 | 0.00% |
| Kolobok-Hydra | 60 | 3,705 | 0.00% |
| Kolobok-T2 | 115 | 6,019 | 0.00% |
| MULE-F | 2 | 75 | 0.00% |
| MULE-MuDR | 721 | 45,686 | 0.01% |
| MULE-NOF | 32 | 2,198 | 0.00% |
| MULE-Ricksha | 1 | 98 | 0.00% |
| Maverick | 2,456 | 1,746,853 | 0.39% |
| Maverick-Mavirus | 3 | 170 | 0.00% |
| Merlin | 42 | 3,223 | 0.00% |
| Novosib | 55 | 5,372 | 0.00% |
| P | 335 | 103,754 | 0.02% |
| P-Fungi | 4 | 218 | 0.00% |
| PIF-HarbS | 3 | 151 | 0.00% |
| PIF-Harbinger | 1,422 | 483,870 | 0.11% |
| PIF-ISL2EU | 67 | 6,695 | 0.00% |
| PIF-Spy | 211 | 127,517 | 0.03% |
| PiggyBac | 738 | 232,007 | 0.05% |
| PiggyBac-A | 2 | 100 | 0.00% |
| PiggyBac-X | 7 | 320 | 0.00% |
| Sola-1 | 522 | 144,591 | 0.03% |
| Sola-2 | 311 | 83,520 | 0.02% |
| Sola-3 | 52 | 3,623 | 0.00% |
| TcMar | 28 | 1,459 | 0.00% |
| TcMar-Ant1 | 4 | 213 | 0.00% |
| TcMar-Cweed | 5 | 213 | 0.00% |
| TcMar-Fot1 | 3,167 | 631,629 | 0.14% |
| TcMar-IS885 | 2 | 91 | 0.00% |
| TcMar-ISRm11 | 41 | 2,979 | 0.00% |
| TcMar-Mariner | 2,668 | 985,961 | 0.22% |
| TcMar-Mogwai | 1 | 62 | 0.00% |
| TcMar-Pogo | 18 | 5,492 | 0.00% |
| TcMar-Sagan | 6 | 267 | 0.00% |
| TcMar-Stowaway | 15 | 563 | 0.00% |
| TcMar-Tc1 | 3,213 | 1,678,720 | 0.38% |
| TcMar-Tc2 | 11 | 674 | 0.00% |
| TcMar-Tc4 | 41 | 2,370 | 0.00% |
| TcMar-Tigger | 91 | 11,832 | 0.00% |
| TcMar-m44 | 319 | 118,786 | 0.03% |
| Zator | 426 | 79,689 | 0.02% |
| Zisupton | 169 | 8,240 | 0.00% |
| hAT | 189 | 15,280 | 0.00% |
| hAT-Ac | 1,788 | 289,101 | 0.06% |
| hAT-Blackjack | 104 | 9,892 | 0.00% |
| hAT-Charlie | 1,403 | 171,605 | 0.04% |
| hAT-Pegasus | 9 | 616 | 0.00% |
| hAT-Restless | 1 | 45 | 0.00% |
| hAT-Tag1 | 159 | 7,129 | 0.00% |
| hAT-Tip100 | 726 | 113,282 | 0.03% |
| hAT-hAT19 | 15 | 1,254 | 0.00% |
| hAT-hAT5 | 5 | 353 | 0.00% |
| hAT-hATm | 123 | 23,558 | 0.01% |
| hAT-hATw | 15 | 801 | 0.00% |
| hAT-hATx | 75 | 7,079 | 0.00% |
| hAT-hobo | 17 | 1,074 | 0.00% |
| - | 2,134 | 410,757 | 0.09% |
|  |  |  |  |
| LINE |  |  |  |
| CR1 | 2,847 | 1,028,934 | 0.23% |
| CR1-Zenon | 156,703 | 25,881,945 | 5.79% |
| CRE | 213 | 20,571 | 0.00% |
| CRE-Ambal | 4 | 294 | 0.00% |
| CRE-Odin | 12 | 566 | 0.00% |
| Deceiver | 4 | 183 | 0.00% |
| Dong-R4 | 1,761 | 513,981 | 0.12% |
| Dualen | 1 | 49 | 0.00% |
| I | 1,570 | 982,185 | 0.22% |
| I-Jockey | 931 | 454,363 | 0.10% |
| L1 | 615 | 32,699 | 0.01% |
| L1-DRE | 3 | 132 | 0.00% |
| L1-Tx1 | 194 | 11,281 | 0.00% |
| L1-Zorro | 2 | 37 | 0.00% |
| L2 | 50,039 | 12,847,712 | 2.88% |
| Penelope | 6,868 | 1,054,848 | 0.24% |
| Proto1 | 4 | 224 | 0.00% |
| Proto2 | 648 | 258,295 | 0.06% |
| R1 | 87,593 | 17,001,747 | 3.81% |
| R1-LOA | 130 | 9,883 | 0.00% |
| R2 | 45 | 2,214 | 0.00% |
| R2-Hero | 8 | 316 | 0.00% |
| R2-NeSL | 21 | 1,100 | 0.00% |
| RTE | 1 | 34 | 0.00% |
| RTE-BovB | 2,946 | 924,669 | 0.21% |
| RTE-ORTE | 12 | 452 | 0.00% |
| RTE-RTE | 28,421 | 4,657,170 | 1.04% |
| RTE-X | 111 | 7,192 | 0.00% |
| Rex-Babar | 61 | 2,615 | 0.00% |
| Tad1 | 52 | 2,636 | 0.00% |
| - | 361 | 448,789 | 0.10% |
|  |  |  | 14.81% |
| LTR |  |  |  |
| Cassandra | 1 | 51 | 0.00% |
| Caulimovirus | 124 | 6,515 | 0.00% |
| Copia | 2,305 | 988,450 | 0.22% |
| DIRS | 306 | 106,056 | 0.02% |
| DIRS-Q | 3 | 141 | 0.00% |
| ERV-Foamy | 4 | 267 | 0.00% |
| ERV-Lenti | 1 | 44 | 0.00% |
| ERV1 | 383 | 20,019 | 0.00% |
| ERV4 | 29 | 1,638 | 0.00% |
| ERVK | 215 | 11,438 | 0.00% |
| ERVL | 22 | 1,092 | 0.00% |
| ERVL-MaLR | 5 | 251 | 0.00% |
| Gypsy | 7,604 | 9,553,236 | 2.14% |
| Ngaro | 67 | 10,439 | 0.00% |
| Pao | 2,399 | 1,941,066 | 0.43% |
| Viper | 1 | 72 | 0.00% |
| - | 6,352 | 1,206,185 | 0.27% |
|  |  |  |  |
| Other |  |  |  |
| DNA_virus | 1 | 50 | 0.00% |
|  |  |  |  |
| RC |  |  |  |
| Helitron | 131,317 | 17,655,663 | 3.95% |
| Helitron-2 | 5 | 110 | 0.00% |
|  |  |  |  |
| Retroposon |  |  |  |
| SVA | 1 | 41 | 0.00% |
| - | 484 | 32,693 | 0.01% |
|  |  |  |  |
| SINE |  |  |  |
| 5S | 1 | 16 | 0.00% |
| 5S-Deu | 45 | 3,400 | 0.00% |
| 5S-RTE | 8 | 445 | 0.00% |
| 5S-Sauria-RTE | 1 | 16 | 0.00% |
| 7SL | 3 | 572 | 0.00% |
| B2 | 1 | 50 | 0.00% |
| B4 | 3 | 129 | 0.00% |
| ID | 3 | 152 | 0.00% |
| MIR | 3 | 196 | 0.00% |
| U | 5 | 126 | 0.00% |
| tRNA | 51,555 | 7,644,572 | 1.71% |
| tRNA-5S | 3 | 339 | 0.00% |
| tRNA-CR1 | 5 | 251 | 0.00% |
| tRNA-Core | 6 | 273 | 0.00% |
| tRNA-Core-RTE | 1 | 76 | 0.00% |
| tRNA-Deu | 130 | 6,971 | 0.00% |
| tRNA-Deu-I | 1 | 0 | 0.00% |
| tRNA-Deu-L2 | 4 | 270 | 0.00% |
| tRNA-I | 1 | 94 | 0.00% |
| tRNA-L2 | 14 | 584 | 0.00% |
| tRNA-Meta | 6 | 396 | 0.00% |
| tRNA-R2 | 1 | 32 | 0.00% |
| tRNA-RTE | 22,176 | 3,636,607 | 0.81% |
| tRNA-V | 3 | 171 | 0.00% |
| tRNA-V-RTE | 2 | 25 | 0.00% |
|  |  |  |  |
| Unknown |  |  |  |
| centromeric | 1 | 45 | 0.00% |
| - | 174,622 | 26,326,272 | 5.89% |
|  |  |  |  |
| total interspersed | 769,378 | 143,300,294 | 32.07% |
|  |  |  |  |
| Low_complexity | 14,784 | 699,285 | 0.16% |
| Satellite | 1,941 | 150,474 | 0.03% |
| Y-chromosome | 1 | 43 | 0.00% |
| acro | 6 | 277 | 0.00% |
| macro | 4 | 199 | 0.00% |
| Simple_repeat | 80,347 | 3,720,653 | 0.83% |
| rRNA | 74 | 60,536 | 0.01% |
| scRNA | 1 | 6 | 0.00% |
| snRNA | 108 | 11,837 | 0.00% |
| srpRNA | 1 | 275 | 0.00% |
| tRNA | 457 | 29,176 | 0.01% |
|  |  |  |  |
| Total | 867,102 | 147,973,055 | 33.12% |
