# Supplementary material for: Genome mapping coupled with CRISPR gene editing reveals a P450 gene confers avermectin resistance in the beet armyworm
Source: PLoS Genet. 2021 Jul 12;17(7):e1009680. doi: 10.1371/journal.pgen.1009680 (PMC8297932; doi:10.1371/journal.pgen.1009680)
Supplement: S4 Table — (DOCX) [file pgen.1009680.s012.docx]

**S4 Table. Primers for template DNA for in vitro transcription of sgRNAs and amplifying genomic DNA fragment of CYP9A genes.**

| **Name** | **Sequence (5’>3’)** |
| --- | --- |
| A40-sgRNA-F | TAATACGACTCACTATAGGTGATGGACTTGCTGTTC |
| A40-sgRNA-R | TTCTAGCTCTAAAACGAACAGCAAGTCCATCAC |
| A107-sgRNA-F | TAATACGACTCACTATAGGGTAAACCGAACCCGAAAG |
| A107-sgRNA-R | TTCTAGCTCTAAAACCTTTCGGGTTCGGTTTACC |
| A98-sgRNA-F | TAATACGACTCACTATAGGCCACAATCTTAATACCTC |
| A98-sgRNA-R | TTCTAGCTCTAAAACGAGGTATTAAGATTGTGGC |
| A186-sgRNA-F1 | TAATACGACTCACTATAGATTTATACAATGCCTTCC |
| A186-sgRNA-R1 | TTCTAGCTCTAAAACGGAAGGCATTGTATAAAT |
| A40F | CTTCTCTCTGTTTTTCATTGTGCC |
| A40R | CATTCATGGAAGAAGTTTGCTTG |
| A107F | GTATTCTTCATTGCTGGATTTG |
| A107R | GAGACCGCGCTTGTTTGAAC |
| A98F | GAACGTTTGGTGCAGGAGAT |
| A98R | TAAGTTGAAACTATCCGTGGCTAG |
| A9F | GCCCTTCGGTGTTGGACCT |
| A9R | CCAGTGTCCTCCTTTCAGCCT |
| A186F1 | CTTCAAGCCCATTCCTCTACTG |
| A186R1 | GCGACCTAATTACGAGCCTCAG |
| A186F2 | CATTGTTGATATATTTATTATAGTTCCTAG |
| A186R2 | CAGAACTTTTGTTTATTAATGTTTTC |
