# Supplementary material for: Genome mapping coupled with CRISPR gene editing reveals a P450 gene confers avermectin resistance in the beet armyworm
Source: PLoS Genet. 2021 Jul 12;17(7):e1009680. doi: 10.1371/journal.pgen.1009680 (PMC8297932; doi:10.1371/journal.pgen.1009680)
Supplement: S5 Table — (DOCX) [file pgen.1009680.s013.docx]

**S5 Table. Mutagenesis induced by gRNA/Cas9.**

| Strain | No. of injected embryo | Hatched rate (%) | Pupation rate (%) | Rate of positive single pairs in G_0_ | No. of positive single pairs inherited deletion event in G_1_ | Rate of heterozygous for the deletion event in G_1_ | Rate of homozygote for the deletion event in G_2_ |
| --- | --- | --- | --- | --- | --- | --- | --- |
| WH-EB-dA40-A98 | 435 | 49.0% (213/435) | 61.5% (131/213) | 26.7% (8/30) | 3 | ♀: 3.3% (1/30) | ♀: 20.8% (5/24) |
|  |  |  |  |  |  | ♂: 10.0% (3/30 ) | ♂: 8.3% (2/24 ) |
| WH-EB-dA40-A107 | 434 | 51.8% (225/434) | 80.9% (182/225) | 11.5% (9/78) | 5 | ♀: 40.0% (12/30) | ♀: 15.9% (7/44) |
|  |  |  |  |  |  | ♂: 43.3% (13/30 ) | ♂: 9.1% (4/44 ) |
| WH-EB-dA107-A98 | 348 | 33.6% (147/348) | 66.0% (97/147) | 16.7% (5/30) | 4 | ♀: 10.0% (3/30) | ♀: 16.7% (4/24) |
|  |  |  |  |  |  | ♂: 13.3% (4/30 ) | ♂: 12.5 (3/24 ) |
| WH-EB-A186-KO | 205 | 27.8% (57/205) | 84.2% (48/57) | - | - | - | ♀: 25.0% (3/12) |
|  |  |  |  |  |  |  | ♂: 25.0% (3/12 ) |
