# Supplementary material for: Genome mapping coupled with CRISPR gene editing reveals a P450 gene confers avermectin resistance in the beet armyworm
Source: PLoS Genet. 2021 Jul 12;17(7):e1009680. doi: 10.1371/journal.pgen.1009680 (PMC8297932; doi:10.1371/journal.pgen.1009680)
Supplement: S6 Table — (DOCX) [file pgen.1009680.s014.docx]

**S6 Table. Primers for amplifying *SeCYP9A* subfamily genes and analysis *SeCYP9A* subfamily genes expression.**

| Name | Sequence (5’>3’) |
| --- | --- |
| full-A107F | ATGATTATCCTCTCAGTCTGGTTGG |
| full-A107R | CTAAGTCCTTGCCCTGAACTTCAGCC |
| full-A27F | ATGATAATATGGATTCTCTGTGCGG |
| full-A27R | TCACTTTCTAGGTCTAACTCTAAGCC |
| full-A11F | ATGATTATCTTTTTCATTTGGTTGG |
| full-A11R | TTATTTTCTTTGTCTAAATCTAAGCC |
| full-A98F | ATGATCCTCACGCTAATATGGGTGG |
| full-A98R | CTATTTTCTTAGCCTACACCTTAACC |
| full-A186F | ATGATAATACTGCTGATTTGGGTGAC |
| full-A186R | CTATTTTCTCAGTCGGAATCTAAGC |
| qCYP9A107-F | GGGTAAACCGAACCCGAAAG |
| qCYP9A107-R | GGTCCAACACCGAAGGGCAT |
| qCYP9A27-F | TGGGCAAACCAAATAAGGGTT |
| qCYP9A27-R | AATTCCGAAGGGGATGTAGGC |
| qCYP9A11-F | TGTCGGGCCAAGAAACTGC |
| qCYP9A11-R | TCTAAATCTAAGCCAGTGTCCTCCTT |
| qCYPA186-F | TCTGTTGGATTACGGAAAGTCGTC |
| qCYPA186-R | GTTGAAGTCAAACTTGCCACCATT |
| qCYP9A98-F | CTAATAAAGTTTGGACCGACGATG |
| qCYP9A98-R | CCACCTTTCCACCAAGTTTCTC |
| qGADPHF | AACATTTATCTCTACAACGCAATC |
| qGADPHR | GTGACAACCACTCATCTATCTTC |
| β-actinF | AGCGTGACATCAAGAGGACT |
| β-actinR | CTCCATGTATGCCTGCTTCG |
