# Supplementary material for: Genome mapping coupled with CRISPR gene editing reveals a P450 gene confers avermectin resistance in the beet armyworm
Source: PLoS Genet. 2021 Jul 12;17(7):e1009680. doi: 10.1371/journal.pgen.1009680 (PMC8297932; doi:10.1371/journal.pgen.1009680)
Supplement: S7 Table — (DOCX) [file pgen.1009680.s015.docx]

**S7 Table. Analysis parameters for substrates and metabolites detection.**

| Substrate | Parent ion (m/z) | Daughter ions (m/z) | Cone votage (V) | Collision energy (V) |
| --- | --- | --- | --- | --- |
| Emamectin benzoate B1a | 886.8 | 158.2* | 35 | 33 |
|  |  | 82.1 | 35 | 45 |
| Abamectin B1a | 890.8 | 305.3* | 35 | 17 |
|  |  | 567.6 | 35 | 10 |
| Hydroxy-Emamectin benzoate B1a | 903 | 158.2* | 20 | 40 |
|  |  | 126.1 | 20 | 40 |
| O-desmethyl-Emamectin benzoate B1a | 873 | 158.2* | 20 | 40 |
|  |  | 288.1 | 20 | 40 |
| Hydroxy-Abamectin B1a | 906.7 | 305.3* | 20 | 35 |
|  |  | 567.5 | 20 | 35 |
| O-desmethyl- Abamectin B1a | 876.7 | 305.2* | 20 | 35 |
|  |  | 567.3 | 20 | 35 |

* quantitative daughter ions.
